# Intensive training of motor function and functional skills among young children with cerebral palsy: a systematic review and meta-analysis

**DOI:** 10.1186/s12887-014-0292-5

**Published:** 2014-12-05

**Authors:** Hilde Tinderholt Myrhaug, Sigrid Østensjø, Lillebeth Larun, Jan Odgaard-Jensen, Reidun Jahnsen

**Affiliations:** Faculty of Health Sciences, Oslo and Akershus University College of Applied Sciences, St. Olavs plass, Postbox 4, 0130 Oslo, Norway; Primary Health Care Unit, Norwegian Knowledge Centre for the Health Services, St. Olavs plass, Postbox 7004, 0130 Oslo, Norway; Global Health Unit, Norwegian Knowledge Centre for the Health Services, St. Olavs plass, Postbox 7004, 0130 Oslo, Norway; Department of Clinical Neuroscience for Children, Oslo University Hospital, Rikshospitalet, Postbox 4950, Nydalen, 0424 Oslo Norway

**Keywords:** Young children, Cerebral palsy, Intensive training, Motor function, Functional skills, Systematic review

## Abstract

**Background:**

Young children with cerebral palsy (CP) receive a variety of interventions to prevent and/or reduce activity limitations and participation restrictions. Some of these interventions are intensive, and it is a challenge to identify the optimal intensity. Therefore, the objective of this systematic review was to describe and categorise intensive motor function and functional skills training among young children with CP, to summarise the effects of these interventions, and to examine characteristics that may contribute to explain the variations in these effects.

**Methods:**

Ten databases were searched for controlled studies that included young children (mean age less than seven years old) with CP and assessments of the effects of intensive motor function and functional skills training. The studies were critically assessed by the Risk of bias tool (RoB) and categorised for intensity and contexts of interventions. Standardised mean difference were computed for outcomes, and summarised descriptively or in meta-analyses.

**Results:**

Thirty-eight studies were included. Studies that targeted gross motor function were fewer, older and with lower frequency of training sessions over longer training periods than studies that targeted hand function. Home training was most common in studies on hand function and functional skills, and often increased the amount of training. The effects of constraint induced movement therapy (CIMT) on hand function and functional skills were summarised in six meta-analyses, which supported the existing evidence of CIMT. In a majority of the included studies, equal improvements were identified between intensive intervention and conventional therapy or between two different intensive interventions.

**Conclusions:**

Different types of training, different intensities and different contexts between studies that targeted gross and fine motor function might explain some of the observed effect variations. Home training may increase the amount of training, but are less controllable. These factors may have contributed to the observed variations in the effectiveness of CIMT. Rigorous research on intensive gross motor training is needed.

**Systematic review registration number:**

CRD42013004023.

**Electronic supplementary material:**

The online version of this article (doi:10.1186/s12887-014-0292-5) contains supplementary material, which is available to authorized users.

## Background

All young children, including children with cerebral palsy (CP) develop basic motor function and learn a variety of functional skills during their first years of life [1,2]. However, children with CP need more support in this developmental process, and therefore receive a variety of interventions with different intensities and diverse results on activity and participation [3-5]. It is a challenge to identify the optimal intensity of these interventions.

Research on intensive interventions of gross motor function and functional skills is limited, inadequately described, and its effects are inconclusive [6]. In contrast, the body of evidence targeting hand function has shown promising results [4,8-10]. A review from 2014 [10] showed that constraint induced movement therapy (CIMT) led to better hand function compared with conventional therapy. When CIMT was compared at an equal intensity of bimanual training, both intervention groups showed similar improvements in hand function [8,10]. Earlier systematic reviews included children with a wide age range [4,7,10]. In children with CP, intensive intervention before the age of seven is recommended for optimizing motor function and learning functional skills, because from a maturational and neuroplasticity perspective the greatest gains will be made during this window [1,2,11].

Intensive interventions for children with CP refer to the frequency and amount of training, the duration of the training session (minutes or hours), and the duration of the training period (weeks or months) [12,13]. The studies included in the systematic reviews of physiotherapy (PT) often define intensity as the frequency of therapy or training sessions [5,7]. Arpino et al. [6] operationally defined any treatment provided more than three times per week as intensive. However, Sakzewski et al. [10] used both the frequency and duration of each session to describe the intensity of therapy. Physiotherapy sessions are typically offered 1–2 times per week to young children with CP as reported in Norway, Canada and the US [14,15]. Therefore, we chose to define intensive training as more than two times per week.

In an editorial commentary, Palisano and Murr made a distinction between intensive interventions, which was defined by the frequency of therapy sessions, and the practice of activities in natural environments [12]. Home practice has been shown to augment and increase the amount of training [10]. However, compliance is a challenge. It has been reported that parents taught to carry out a therapist set program in home environments are less compliant compared with parents taught to use everyday activities as learning opportunities [16,17]. The optimal intensity in relation to the type, setting, and organisation of the intervention is a concern and requires further exploration.

The aim of this systematic review was to describe and categorise intensive motor function and functional skills training among young children with CP, and to summarise the effects of these interventions. Systematic descriptions will allow comparisons of the characteristics of the different types of interventions, as well as the investigation of characteristics that may explain the observed variations in effects.

## Methods

The protocol of this systematic review was registered in PROSPERO table with registration number CRD42013004023. Ethical approval was not required.

### Search strategy

MEDLINE, Embase, PsycINFO, Cochrane Library, ERIC, OT Seeker, Cinahl, ISI Web of Science, SveMed+, and PEDro were searched in October 2012. The search strategy used free text word and subject headings adapted to each database. The full electronic search strategy for Ovid MEDLINE(R) is found in Additional file 1. The reference lists of relevant systematic reviews were also manually searched. An updated search was conducted in the Cochrane Central Register of Controlled Trials (Central), PEDro and ISI Web of science in September 2014. A list of included studies of awaiting assessment is attached (Additional file 2).

### Selection criteria

We included trials with the following criteria: (a) a study population of CP with a mean age less than seven years; (b) evaluated the effects of motor function (e.g., mobility and grasping) and functional skills training (e.g., eating and playing) performed three times or more per week at the clinic, in the kindergarten, or at home; (c) was compared to another intervention (e.g., conventional therapy), the same type of intervention provided less frequently, or another intensive intervention; and (d) with outcomes in the activity and participation components of the ICF [3], measured as hand function, gross motor function, and/or functional skills. In addition, the included studies were required to be controlled trials, published in peer review journals in the period from 1948 to October 2012 in English or a Scandinavian language. Studies were excluded if the training was combined with passive interventions (e.g., botulinum toxin-A (BoNT) injections, massage, or neuromuscular stimulation), or if the outcomes were only within the body functions and structures component of the ICF (e.g., range of motion and spasticity).

### Selection of studies and data extraction

All steps in the selection and extraction processes (i.e., the study selection, data extraction, and risk of bias evaluation) were assessed independently by two reviewers. Any disagreement between the reviewers in these processes was resolved by discussions with the group of authors. The titles and abstracts of all retrieved references were screened. The full texts of relevant publications were reviewed and were included if they met the inclusion criteria. The data from the included studies were extracted using a piloted data extraction form, which included information on the study population, design, interventions, comparison, outcome measures, and results (Additional file 3). Authors of included studies were not contacted for missing data.

### Risk of bias

The risk of bias tool [18] includes the following items: sequence generation, allocation concealment, integrity of blinding, the completeness of outcome data, selective reporting, and other potential sources of bias. The items in the risk of bias assessment were classified according to the extent to which bias was prevented and included ratings of low, high, or unclear. An overall assessment of the risk of bias was assigned to each included study as suggested in the Cochrane Handbook [18]. When five items were assessed as a low risk of bias within a study, the study was assigned an overall low risk of bias. This characterisation indicates that bias is unlikely to affect the results.

### Data analysis

Intervention characteristics were categorised according to the outcome (hand function, gross motor function, and functional skills), intensity (amount and duration of training), and context of intervention (setting, organisation, goals, and parental involvement) (Table 1). The intensity of training was described as the amount of training and duration of the training periods. The amount was categorised into four groups according to frequency of sessions and use of home training: (1) 2–7 training sessions per week with additional home training, (2) 3–7 training sessions per week, (3) training more than one hour per day, and (4) training more than one hour per day with additional home training (Table 1). The duration was categorised as ≤ four weeks, 5–12 weeks, or >12 weeks. The characteristics were coded as met or not met.

**Table 1:** **Characteristics of the included interventions (Ѵ = characteristic is present)**

	***Intensity***												***Context of intervention***											
			***Amount***				***Duration***			***Setting***			***Organisation***				***Goals***					***Parent involvement***		
***Study***	***N=***	***Outcome***	Sessions* ***2-7/ wk + home training***	Sessions* 3–7 /wk	***> 1 hr/day***	***>1 hr/day*** ***+ home training***	***≤ 4 wks***	***5-12*** ***wks***	***>12 wks***	***Home***	***Kindergarten***	***Clinic***	***Individual***	***Group***	***Home program***	***In daily activities *** ***at home***	***General***	***Specific***	***Parent set***	***Therapist *** ***set***	***Shared set***	***Facilitator***	***Performer***	***Parent-directed training***
*Al-Oraibi* [19]	*14*	*HF*				*Ѵ*		*Ѵ*		*Ѵ*		*Ѵ*	*Ѵ*		*Ѵ*								*Ѵ*	*Ѵ*
*Aarts* [20,21]	*52*	*HF,FS*	*Ѵ*					*Ѵ*		*Ѵ*		*Ѵ*	*Ѵ*	*Ѵ*		*Ѵ*		*Ѵ*	*Ѵ*			*Ѵ*		*Ѵ*
			*Ѵ*					*Ѵ*		*Ѵ*	*Ѵ*	*Ѵ*	*Ѵ*		*Ѵ*								*Ѵ*	*Ѵ*
*Eliasson* [22]	*25*	*HF*			*Ѵ*			*Ѵ*		*Ѵ*	*Ѵ*		*Ѵ*		*Ѵ*								*Ѵ*	*Ѵ*
*Facchin* [23]	*105*	*HF*	*Ѵ*					*Ѵ*		*Ѵ*		*Ѵ*	*Ѵ*		*Ѵ*		*Ѵ*			*Ѵ*			*Ѵ*	*Ѵ*
			*Ѵ*					*Ѵ*		*Ѵ*		*Ѵ*	*Ѵ*		*Ѵ*		*Ѵ*			*Ѵ*			*Ѵ*	*Ѵ*
*Gordon* [24]	*42*	*HF*				*Ѵ*	*Ѵ*			*Ѵ*		*Ѵ*	*Ѵ*	*Ѵ*	*Ѵ*			*Ѵ*	*Ѵ*				*Ѵ*	*Ѵ*
*Smania* [25]	*10*	*HF*				*Ѵ*		*Ѵ*		*Ѵ*	*Ѵ*	*Ѵ*	*Ѵ*		*Ѵ*	*Ѵ*						*Ѵ*		*Ѵ*
*Charles* [26]	*22*	*HF*				*Ѵ*	*Ѵ*			*Ѵ*		*Ѵ*	*Ѵ*	*Ѵ*	*Ѵ*							*Ѵ*		
*Eliasson* [27]	*41*	*HF*			*Ѵ*			*Ѵ*		*Ѵ*	*Ѵ*	*Ѵ*	*Ѵ*			*Ѵ*							*Ѵ*	*Ѵ*
*Law* [28]	*72*	*HF*	*Ѵ*						*Ѵ*	*Ѵ*		*Ѵ*	*Ѵ*		*Ѵ*		*Ѵ*						*Ѵ*	*Ѵ*
*Case-Smith* [29]	*18*	*HF,FS*			*Ѵ*		*Ѵ*			*Ѵ*	*Ѵ*		*Ѵ*			*Ѵ*						*Ѵ*		*Ѵ*
*Hsin* [30]	*22*	*HF,FS*	*Ѵ*				*Ѵ*			*Ѵ*			*Ѵ*		*Ѵ*	*Ѵ*						*Ѵ*		
*Rostami* [31]	*14*	*HF,*	*Ѵ*				*Ѵ*			*Ѵ*	*Ѵ*		*Ѵ*		*Ѵ*	*Ѵ*		*Ѵ*	*Ѵ*				*Ѵ*	*Ѵ*
		*FS*	*Ѵ*				*Ѵ*					*Ѵ*	*Ѵ*		*Ѵ*			*Ѵ*	*Ѵ*				*Ѵ*	*Ѵ*
*Lin* [32]	*22*	*HF,FS*	*Ѵ*				*Ѵ*			*Ѵ*			*Ѵ*			*Ѵ*						*Ѵ*		*Ѵ*
			*Ѵ*				*Ѵ*			*Ѵ*			*Ѵ*			*Ѵ*						*Ѵ*		
*Taub* [33]	*20*	*HF,FS*			*Ѵ*		*Ѵ*			*Ѵ*	*Ѵ*		*Ѵ*		*Ѵ*	*Ѵ*							*Ѵ*	*Ѵ*
*Wallen* [34]	*50*	*HF,FS*	*Ѵ*					*Ѵ*		*Ѵ*	*Ѵ*	*Ѵ*	*Ѵ*		*Ѵ*	*Ѵ*		*Ѵ*	*Ѵ*				*Ѵ*	*Ѵ*
			*Ѵ*					*Ѵ*		*Ѵ*	*Ѵ*	*Ѵ*	*Ѵ*			*Ѵ*		*Ѵ*	*Ѵ*				*Ѵ*	*Ѵ*
*De Luca* [35]*, Taub* [36]	*18*	*HF, FS*			*Ѵ*		*Ѵ*					*Ѵ*	*Ѵ*											*Ѵ*
					*Ѵ*		*Ѵ*			*Ѵ*		*Ѵ*	*Ѵ*				*Ѵ*		*Ѵ*			*Ѵ*		*Ѵ*
*Law* [37]	*52*	*HF,FS*	*Ѵ*						*Ѵ*	*Ѵ*		*Ѵ*	*Ѵ*		*Ѵ*		*Ѵ*						*Ѵ*	*Ѵ*
*Brandao [60 ]*	*16*	*HF,FS*				*Ѵ*	*Ѵ*			*Ѵ*		*Ѵ*	*Ѵ*		*Ѵ*							*Ѵ*		*Ѵ*
*Sung* [38]	*31*	*HF,FS*	*Ѵ*					*Ѵ*		*Ѵ*		*Ѵ*	*Ѵ*			*Ѵ*		*Ѵ*				*Ѵ*		*Ѵ*
*Carlsen* [39]	*20*	*HF,GM*	*Ѵ*					*Ѵ*		*Ѵ*		*Ѵ*	*Ѵ*	*Ѵ*	*Ѵ*	*Ѵ*							*Ѵ*	*Ѵ*
*Choi* [40]	*10*	*GM*		*Ѵ*				*Ѵ*				*Ѵ*	*Ѵ*											
*Kwon* [41]	*32*	*GM*		*Ѵ*				*Ѵ*				*Ѵ*	*Ѵ*											
*Shamsodini* [42]	*27*	*GM*		*Ѵ*				*Ѵ*				*Ѵ*		*Ѵ*										
				*Ѵ*				*Ѵ*		*Ѵ*			*Ѵ*										*Ѵ*	*Ѵ*
*Christiansen* [43]	*25*	*GM*		*Ѵ*					*Ѵ*			*Ѵ*	*Ѵ*										*Ѵ*	*Ѵ*
*Lee* [44]	*17*	*GM*		*Ѵ*				*Ѵ*				*Ѵ*	*Ѵ*											
*Kanda* [45]	*10*	*GM*	*Ѵ*						*Ѵ*	*Ѵ*		*Ѵ*	*Ѵ*		*Ѵ*								*Ѵ*	*Ѵ*
*Bower* [46]	*56*	*GM*		*Ѵ*					*Ѵ*			*Ѵ*	*Ѵ*					*Ѵ*			*Ѵ*			
				*Ѵ*					*Ѵ*			*Ѵ*	*Ѵ*				*Ѵ*			*Ѵ*				
*Bower* [47]	*44*	*GM*		*Ѵ*			*Ѵ*					*Ѵ*	*Ѵ*				*Ѵ*			*Ѵ*				
				*Ѵ*			*Ѵ*					*Ѵ*	*Ѵ*					*Ѵ*			*Ѵ*			
Sherzer [48]	24	GM	*Ѵ*						*Ѵ*	*Ѵ*		*Ѵ*	*Ѵ*		*Ѵ*								*Ѵ*	*Ѵ*
Weindling [49]	88	GM,FS	*Ѵ*						*Ѵ*	*Ѵ*		*Ѵ*	*Ѵ*		*Ѵ*		*Ѵ*					*Ѵ*		*Ѵ*
Løwing [50]	44	GM,FS	*Ѵ*					*Ѵ*		*Ѵ*	*Ѵ*	*Ѵ*	*Ѵ*	*Ѵ*		*Ѵ*		*Ѵ*			*Ѵ*		*Ѵ*	*Ѵ*
Hur [51]	40	GM,FS		*Ѵ*					*Ѵ*			*Ѵ*		*Ѵ*										
				*Ѵ*					*Ѵ*		*Ѵ*			*Ѵ*										
Brandao [52]	16	FS				*Ѵ*	*Ѵ*			*Ѵ*		*Ѵ*	*Ѵ*	*Ѵ*	*Ѵ*			*Ѵ*	*Ѵ*				*Ѵ*	
Dalvand [53]	45	FS		*Ѵ*				*Ѵ*				*Ѵ*	*Ѵ*											
				*Ѵ*				*Ѵ*				*Ѵ*		*Ѵ*										
				*Ѵ*				*Ѵ*				*Ѵ*		*Ѵ*										*Ѵ*
McConahie [54]	85	FS		*Ѵ*					*Ѵ*			*Ѵ*		*Ѵ*		*Ѵ*							*Ѵ*	*Ѵ*
Stiller [55]	21	HF,GM,		*Ѵ*				*Ѵ*				*Ѵ*	*Ѵ*	*Ѵ*										
		FS		*Ѵ*				*Ѵ*				*Ѵ*	*Ѵ*	*Ѵ*										
				*Ѵ*				*Ѵ*				*Ѵ*	*Ѵ*	*Ѵ*										
Reddihough [56]	34	HF,GM,		*Ѵ*					*Ѵ*			*Ѵ*	*Ѵ*	*Ѵ*										
		FS		*Ѵ*					*Ѵ*			*Ѵ*						*Ѵ*						
Coleman [57]	26	HF,GM,FS		*Ѵ*					*Ѵ*	*Ѵ*	*Ѵ*	*Ѵ*		*Ѵ*		*Ѵ*								

*One sessions = 30-60 minutes, HF (hand function), GM (gross motor function), and FS (functional skills).

Standardised mean differences (SMD) were computed for outcomes based on post treatment mean scores for the study groups, except for studies that showed clinically or statistically significant baseline differences or where the post treatment mean scores were not reported. The results from these studies were not calculated, due to lack of information. Review Manager Software (RevMan5; Cochrane Information Management System) was used to compute the SMD and to summarise statistically randomised controlled data if the included studies were comparable in terms of the type of training, amount of training, and outcomes. In the meta-analyses, the outcomes were categorised as unimanual or bimanual hand function, gross motor function, and functional skills. A random effects model was used to account for pooling effects due to the clinical heterogeneity of the included studies. Double-data entries were performed. We aimed to examine characteristics that may have contributed to explain the variations in effects. However, the meta-regression analyses could not be performed because of the small number of studies and the clinical heterogeneity between studies.

## Results

**Figure 1:**
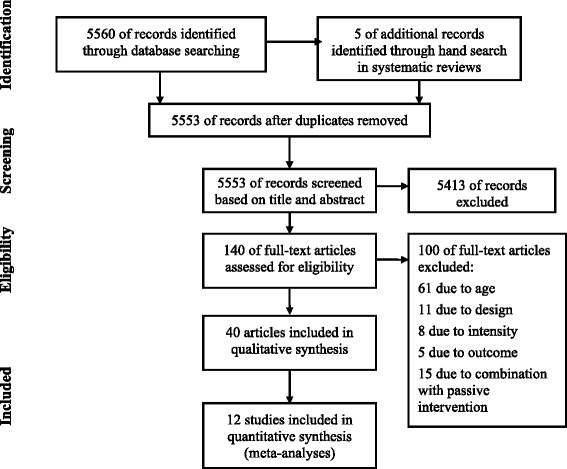
**Selection of studies.**

The results of the search strategy are shown in Figure 1. The search yielded 5,553 unique references, of which, 5,413 references were excluded based on the screening of their titles and abstracts; 140 articles were reviewed in full text. Forty articles, which corresponded to 38 studies from Asia (n = 12), Australia (n = 3), Europe (n = 11), and North America (n = 12), were included.

An overview of the included studies is presented in Additional file 4. The 38 studies included 1407 children with all levels of gross and fine motor function [58,59]. The studies utilised 31 assessment tools, which are described in Additional file 4.

**Figure 2:**
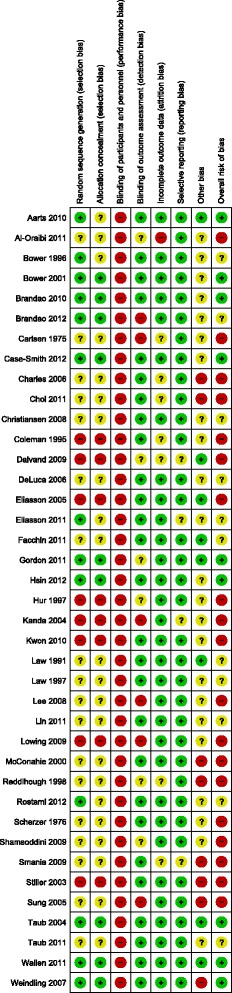
**Risk of bias.**

Twenty-nine studies were randomised controlled studies, and nine studies were controlled before and after studies. The risk of bias within studies is shown in Figure 2. Nine studies had a low risk of bias [20,21,24,29,30,34,36,46,49,60], 11 articles of 10 studies had an unclear risk of bias [22,23,28,31-33,35,37,43,47,52], and 19 studies had a high risk of bias [19,25-27,38-42,44,45,48,50,51,53-57].

### Characteristics of interventions

The characteristics of the intensive interventions included in this systematic review are coded and shown in Table 1. The interventions were categorised according to the outcome, intensity, and context of interventions. Interventions reported as conventional therapy, usual care, conventional paediatric treatment and standard care refer to interventions performed less than three times per week and the type of training was seldom described and not categorised in Table 1.

#### Characteristics of interventions that aimed to improve hand function

Of the 23 studies that reported outcomes for hand function, seven studies reported 2–7 sessions per week with additional home training [20,21,23,30-32,34,38], five studies reported daily training of more than one hour per day [22,27,29,33,35,36], and five studies with a high amount of training (> one hour per day) reported additional home training [19,24-26,60]. Seventeen studies evaluated the effect of constraint induced movement therapy (CIMT), constraint induced therapy (CI), or eco-CI, modified CIT (mCIT), and modified CIMT (mCIMT); hereinafter called CIMT. These 17 CIMT studies were compared with conventional therapy [19,22,25-27,30,33,35,36,38,60], intensive bimanual therapy [20,21,23,24,32,34], more intensive CIMT [29], or intensive training in a different context [31]. The duration of the different CIMT interventions was in all studies less than 12 weeks and took place at the clinic (n = 13) and at home (n = 17). The training was carried out individually (n = 17) and/or as group training sessions (n = 3). Five studies reported therapist set home programs that were incorporated into daily activities [25,30,31,33,34], while six studies reported practices that were only integrated with daily routines of the family [20,21,27,29,32,34,38]. The use of general and specific goals was more prevalent in the studies combined with home training (n = 7) compared with the studies without home training (n = 1). In the studies with home training, all the parents acted as performers or were asked by the therapists to facilitate the child’s everyday skills training at home*.* The parents were offered parent education except in two studies [26,30] (Table 1).

Among the six remaining studies reporting on hand function, three were studies of intensive neurodevelopmental treatment (NDT) [39] and casting [28,37]. These studies included training of hand function over 2–7 sessions per week with additional home training were compared to occupational therapy (OT) [37,39], regular NDT with and without casting [28], and intensive NDT [28]. The intensive NDT lasted more than five weeks and was performed at the clinic and in combination with a home program. Moreover, the training was provided individually (n = 3) and in groups (n = 1). Law [28,37] reported the use of general goals. Parents acted as performers of home training and received supervision. In the remaining three studies [55-57], intensive conductive education (CE) was compared with intensive NDT [56], traditional early intervention program [57], intensive OT and physiotherapy (PT) [55], or intensive special education [55]. The interventions were all performed as 3–7 training sessions per week and lasted 5–12 weeks or more than 12 weeks. Moreover, the training was performed in group training sessions at the clinic, with no home training, defined goals, or parental involvement.

#### Characteristics of interventions that aimed to improve gross motor function

Sixteen studies reported outcomes on gross motor function. Five of these studies reported gross motor function targeted with Vojta training [45], home programs to facilitate motor development [48], goal-directed functional training [50] intensive PT [49], and intensive NDT [39], all performed over 2–7 sessions per week with additional home training. These interventions were compared with non-intensive Vojta treatment [45], traditional passive motion exercises [48], activity-focused training [50], PT and visits by a family support worker (FSWG) [49] or OT [39]. The intensive interventions lasted 5–12 weeks or more than 12 weeks. The training was provided individually (n = 5) and in groups (n = 1) at home and/or at the clinic. Four studies reported therapist set home programs [39,45,48,49], whereas two studies reported practice that was integrated with daily activities [39,50]. Weindling [49] and Løwing [50] used general and specific goals, respectively. Active parental involvement in training, and parent directed training were also reported (n = 5) (Table 1).

The remaining eleven studies that targeted gross motor training were performed 3–7 sessions per week within a task-oriented approach [40], hippotherapy and NDT [41], sensory integration therapy [42], intensive and other types of PT [43,44,46,47] or CE [51,55-57]. These interventions were compared with NDT [40,41,56,57], home program with OT [42], other types of PT [43,44,46,47,55] or intensive special education [51,55]. The training lasted from less than four weeks to more than 12 weeks. It was provided individually (n = 8) and/or in groups (n = 5) only at the clinic. The use of general and specific goals was only reported in two studies [46,47]. Shamsoddini [42] and Christiansen [43] reported parental involvement. The characteristics of CE reported by Hur [51], Stiller [55], Reddihough [56], and Coleman [57] were the same as that described for hand function.

#### Characteristics of interventions that aimed to improve functional skills

Of the 20 studies that reported outcomes on functional skills, nine studies reported 2–7 sessions per week with additional home training [20,21,30-32,34,37,38,49,50], six studies reported training over 3–7 sessions per week [51,53-57], three studies and four articles reported training of more than one hour per day [29,33,35,36], and two studies reported more than one hour of training per day with additional home training [52,60]. The characteristics of these studies are presented in relation to hand or gross motor function, except for the studies by Hur [51], Dalvand [53], McConahie [54], and Brandao [52]. In the studies by Hur [51] and Dalvand [53], the effect of CE performed over 3–7 times per week was compared with intensive special education [51] and NDT or education to parents [53]. Otherwise, the characteristics were similar to the other CE-studies presented earlier. McConahie [54] reported the outcomes of training over 3–7 sessions per week for more than 12 weeks. The intervention was an urban daily mother-child group that took place at the clinic, where the mothers were actively involved and received supervision. In the report by Brandao [52], CIMT was performed for more than one hour per day with additional home training and was compared to intensive bimanual therapy based on functional skills only. The duration of therapy was less than four weeks. The intervention was performed at home with a therapist-set home program and at the clinic in groups. The use of parent set specific goals and parental involvement were also reported.

### Effects on hand function

The results from the 23 studies that targeted hand function are presented in Table 2 or in the meta-analyses [19,21-39,55-57,60], of which, 17 studies evaluated CIMT.

**Table 2:** **Summary of the results**

**Study [ref] (Risk of bias)** ^**A**^	**Outcome (outcome measurement)**	**Treatment duration, wk**	**n**	**Post treatment, ** **mean score (SD)**	**n**	**Post control, ** **mean score (SD)**	SMD (95% CI)*
Aarts [20]	HF (VOAA-DDD, capacity)	8	28	40.5 (29.2)	22	28.6 (28.8)	0.40 (−0.16, 0.97)
Aarts [21]	HF (VOAA-DDD, performance)	8	28	69.6 (21.4)	22	50.4 (28.5)	0.76 (0.18, 1.34)*
(Low)							
Facchin [23]	HF (QUEST)	10	20	72.8	33	72.9	Could not be estimated^B^
(Unclear)	HF (QUEST)	10	19	72.8	33	68.4	Could not be estimated^B^
	HF (Besta Scale, quality of grasp)	10	20	3.15	33	2.88	Could not be estimated^B^
	HF (Besta Scale, quality of grasp)	10	19	3.15	33	3.02	Could not be estimated^B^
Gordon [24]	HF (JTTHF)	3	21	−233.1 (173.8)	21	−249.6 (173.8)	0.09 (−0.51, 0.70)
(Low)							
Smania [25]	HF (Use test)	5	5	NR	5	NR	Could not be estimated^B^
(High)	HF (Function test)	5	5	NR	5	NR	Could not be estimated^B^
Charles [26]	HF (JTTHF)	2	11	−278.5 (240.6)	11	−301.0 (182.2)	0.10 (−0.73, 0.94)
(High)	HF (BOTMP subtest 8)	2	11	7.2 (2.9)	11	5.2 (4.2)	0.53 (−0.32, 1.39)
Law [28]							
(Unclear)	HF (PDMS, fine motor scale)	24	18	28.1 (18.4)	18	30.8 (21.3)	−0.13 (−0.79, 0.52)
	HF (QUEST)	24	18	47.9 (26.8)	18	47.2 (28.9)	0.02 (−0.63, 0.68)
Case-Smith [29]	HF (AHA)	3	9	0.84 (3.3)	9	3.03 (3.9)	−0.58 (−1.53, 0.37)
(Low)	FS (PMAL (QOU))	3	9	3.40 (1.40)	9	3.43 (0.80)	−0.03 (−0.95, 0.90)
Hsin [30]	FS (PMAL (QOU))	4	11	2.6 (0.3)	11	2.3 (0.2)	1.13 (0.22, 2.05)*
(Low)							
Rostami [31]							
(Unclear)	FS (PMAL (QOU))	3.35	7	2.26 (0.29)	7	2.23 (0.30)	0.10 (−0.95, 1.14)
Lin [32]							
	HF (BOTMP-MUE)	4	10	9.00 (5.91)	11	5.77 (6.33)	0.51 (−0.37, 1.38)
(Unclear)	HF (PDMS-grasp)	4	10	45.9 (7.82)	11	44.27(6.23)	0.22 (−0.64, 1.08)
	FS (PMAL (QOU))	4	10	2.84 (0.96)	11	2.26 (0.88)	0.61 (−0.27, 1.49)
Taub [33]	HF (INMAP)	3	10	35.9 (6.2)	10	27.8 (6.6)	1.21 (0.24, 2.18)*
(Unclear)	FS (PMAL)	3	10	3.5 (0.6)	10	1.4 (0.5)	3.64 (2.11, 5.18)*
Wallen [34]	FS (COPM, performance)	8	25	6.1 (2.3)	25	6.0 (1.7)	0.05 (−0.51, 0.60)
(Low)	FS (COMP, satisfaction)	8	25	6.5 (2.4)	25	6.7 (2.2)	−0.09 (−0.64, 0.47)
	FS (PMAL (QOU))	8	25	59.6 (23.6)	25	51.3 (19.7)	0.38 (−0.18, 0.94)
DeLuca [35]	HF (QUEST)	3	9	NR	9	NR	Could not be estimated^B^
(Unclear)	HF (EBS)	3	9	NR	9	NR	Could not be estimated^B^
Taub [36]	FS (PMAL (AOU))	3	9	NR	9	NR	Could not be estimated^B^
(Low)	FS (PMAL (OOL))	3	9	NR	9	NR	Could not be estimated^B^
	HF (EBS)	3	9	21.5 (4.45)	9	15 (5.66)	1.22 (0.19, 2.24)*
	FS (PMAL (QOU))	3	9	2.7 (0.97)	9	1.9 (1.13)	0.72 (−0.24, 1.69)
	HF (TAUT)	3	9	NR	9	NR	Could not be estimated^B^
Law [37]	HF (PDMS, fine motor scale)	16	26	21.8 (8.5)	24	20.9 (9.0)	0.10 (−0.45, 0.66)
(Unclear)	HF (QUEST)	16	26	53.3 (22.9)	24	47.3 (27.7)	0.23 (−0.32, 0.79)
	FS (COPM, performance)	16	26	6.5 (1.6)	24	5.7 (1.4)	0.52 (−0.04, 1.09)
	FS (COPM, satisfaction)	16	26	7.1 (1.9)	24	5.8 (1.8)	0.69 (0.12, 1.26)*
Brandao [60]	FS (PEDI, independence)	2	8	70.25 (8.90)	7	68.37 (3.61)	0.25 (−0.77, 1.27)
(Low)							
Sung [38]	HF (BBT, affected limb)	6	18	10.50 (5.73)	13	9.54 (7.14)	0.15 (−0.57, 0.86)
(High)	HF (BBT, unaffected limb)	6	18	18.12 (10.06)	13	23.15 (17.12)	−0.36 (−1.08, 0.36)
	HF (EDPA)	6	18	7.64 (1.65)	13	7.06 (1.42)	0.36 (−0.36, 1.08)
Carlsen [39]	HF (Denver Development subscales)		6	NR	6	NR	Could not be estimated^B^
(High)	GM (Denver Development subscales)		6	NR	6	NR	Could not be estimated^B^
Choi [40]	GM (GMFM-88, sitting)	6	5	9.4 (3.11)	5	2.0 (2.12)	2.51 (0.63, 4.39)^C^*
(High)							
Kwon [41]	GM (GMFM-66)	8	16	73. 7 (8.3)	16	70.1 (8.1)	0.43 (−0.27, 1.13)
(High)	GM (gait analysis, speed)	8	16	−60.7 (0.1)	16	−68.0 (0.2)	45.01 (−33.21, 56.80)
Shamsoddini [42]	GM (GMFM-88)	12	14	90.1 (11.62)	10	86.3 (7.93)	0.36 (−0.46, 1.18)
(High)							
Christiansen [43]	GM (GMFM-66)	30	10	54.9 (16.5)	14	55.6 (19.7)	−0.04 (−0.85, 0.77)
(Unclear)							
Lee [44]	GM (GMFM-88)	5	9	86.9 (13.4)	8	85.4 (13.5)	0.11 (−0.85, 1.06)
(High)	GM (Gait analysis, speed)	5	9	74.6 (38.7)	8	68.2 (42.9)	Could not be estimated^B^
Kanda [45]	GM (Able to stand or walk 5 sec)	208	5	4	5	0	Could not be estimated^B^
(High)							
Bower [46]	GM (GMFM-88)	24	15	NR	13	NR	Could not be estimated^B^
(Low)							
Bower [47]							
	GM (GMFM-88)	2	22	NR	22	NR	Could not be estimated^B^
(Unclear)							
Scherzer [48]	GM (Motor dev eval form)	24	11	NR	11	NR	Could not be estimated^B^
(High)							
Weindling [49]	GM (GMFM-88)	24	12	50.0 (25.8)	28	45.5 (29.7)	0.15 (−0.52, 0.83)
(Low)	GM (GMFM-88)	24	13	50.0 (25.8)	23	48.0 (30.7)	0.07 (−0.61, 0.75)
	FS (Vineland Daily living)	24	12	25.5 (11.0)	28	24.5 (17.1)	0.07 (−0.47, 0.61)
	FS (Vineland Daily living)	24	13	25.5 (11.0)	22	25.5 (16.3)	0.00 (−0.57, 0.57)
Løwing [50]							
	GM (GMFM-66)	12	22	63.59 (13.15)	22	64.15 (17.33)	−0.04 (−0.63, 0.56)^D^
(High)	FS (PEDI, self-care)	12	22	57.35 (9.40)	22	58.66 (11.63)	−0.12 (−0.71, 0.47)
	FS (PEDI, mobility)	12	22	61.44 (13.89)	22	62.48 (17.75)	−0.06 (−0.66, 0.53)
	FS (Social function)	12	22	61.22 (8.85)	22	63.61 (10.25)	−0.25 (−0.84, 0.35)
Hur [51]	GM (VAB, videotaped, gross motor)	56	19	13.5 (6.2)	17	16.2 (12.4)	−0.27 (−0.93, 0.38)
(High)	GM (Developmental profile2, physical)	56	19	26.5 (13.1)	17	24.7 (11.4)	0.14 (−0.51, 0.80)
	FS (VAB, videotaped, play and leisure)	56	19	39.1 (19.4)	17	51.7 (22.1)	−0.59 (−1.27, 0.08)
	FS (VAB, videotaped, daily living)	56	19	34.1 (14.1)	17	32.3 (10.6)	0.14 (−0.52, 0.80)
	FS (Developmental profile2, self-help)	56	19	50.2 (18.2)	17	42.2 (18.3)	0.43 (−0.23, 1.09)
Brandao [52]	FS (COPM, performance)	3	8	5.54 (1.7)	8	6.58 (1.19)	−0.67 (−1.69, 0.35)
(Unclear)	FS (COMP, satisfaction)	3	8	5.68 (2.06)	8	6.78 (1.64)	−0.56 (−1.56, 0.45)
	FS (PEDI, self-care skills)	3	8	60.12 (6.13)	8	63.5 (5.01)	−0.57 (−1.58, 0.44)
	FS (PEDI, independence)	3	8	29.12 (7.26)	8	31.75 (4.4)	−0.41 (−1.41, 0.58)
Dalvand [53]							
(High)	FS (CDER)	12	7	42.80 (40.04)	15	36.80 (34.42)	0.16 (−0.74, 1.06)
	FS (CDER)	12	8	42.80 (40.04)	15	34.60	0.23 (−0.64, 1.09)
McConahie [54] (High)	FS (IBAS)	80-96	11	−2.75 (1.62)	16	−3.11 (1.10)	0.26 (−0.51, 1.03)
Stiller [55]							
	HF (PDMS, fine motor, grasping)	5	7	1.00 (−1.29)	8	0.25 (1.28)	0.55 (−0.49, 1.59)^C^
(High)	HF (PDMS, fine motor, hand use)	5	7	−2.12 (2.49)	8	−0.28 (1.29)	−0.89 (−1.97, 0.19)^C^
	GM (GMFM-88, lying & rolling)	5	7	1.43 (3.69)	8	0.50 (2.14)	0.30 (−0.73, 1.32)^C^
	GM (GMFM-88, Sitting)	5	7	2.43 (3.10)	8	0.63 (5.07)	0.40 (−0.63, 1.42)^C^
	GM (GMFM-88, crawling & kneeling)	5	7	0.14 (1.57)	8	2.75 (1.91)	−1.39 (−2.56, −0.23)^C^*
	GM (GMFM-88, walking, running, jumping)	5	7	3.29 (4.42)	8	2.63 (4.93)	−0.13 (−1.15, 0.88)^C^
	GM (GMFM-88, standing)	5	7	−1.29 (2.87)	8	0.63 (2.00)	−0.74(−1.80, 0.32)^C^
	FS (PEDI, self-care)	5	7	5.29 (9.55)	8	7.00 (5.55)	−0.21 (−1.23, 0.81)^C^
	FS (PEDI, mobility)	5	7	2.57 (4.04)	8	1.25 (2.60)	0.37 (−0.65, 1.40)^C^
	FS (PEDI, social function)	5	7	4.00 (5.75)	8	5.50 (3.85)	−0.29 (−1.31, 0.73)^C^
Reddihough [56]	HF (VAB, videotaped, fine motor)	24	17	5.15 (2.68)	17	5.47 (3.11)	−0.11 (−0.78, 0.57)
(High)	GM (GMFM-88)	24	9	33.20 (13.82)	13	28.64 (17.83)	0.27 (−0.59, 1.12)
	GM (VAB, videotaped, gross motor)	24	17	6.29 (2.24)	17	5.76 (2.64)	0.21 (−0.46, 0.89)
	FS (VAB, videotaped, feeding)	24	17	5.29 (2.95)	17	4.65 (2.63)	0.22 (−0.45, 0.90)
	FS (VAB, videotaped, play)	24	17	5.87 (3.82)	17	5.14 (3.41)	0.20 (−0.48, 0.87)
	FS (VAB, caregiver reported, feeding)	24	17	5.06 (0.89)	17	4.26 (0.95)	0.85 (0.14, 1.55)*
	FS (VAB, caregiver reported, dressing)	24	17	1.72 (1.62)	17	3.69 (1.42)	−1.26 (−2.01, −0.52)*
	FS (VAB, caregiver reported, play)	24	17	6.31 (0.75)	17	5.78 (1.12)	0.54 (−0.14, 1.23)
	FS (VAB, caregiver reported, toileting)	24	17	3.69 (1.67)	17	3.02 (1.22)	0.45 (−0.23, 1.13)
Coleman [57]	HF (VAB, videotaped, fine motor)	24	11	3.67 (1.87)	9	3.88 (1.97)	−0.11 (−0.99, 0.78)
(High)	GM (VAB, videotaped, gross motor)	24	11	3.53 (1.51)	9	3.76 (1.51)	−0.15 (−1.03, 0.74)
	FS (VAB, videotaped, feeding)	24	11	4.43 (1.65)	9	4.27 (2.16)	0.08 (−0.80, 0.96)
	FS (VAB, Caregiver reported, feeding)	24	11	2.75 (1.68)	9	3.36 (1.57)	−0.36 (−1.25, 0.53)
	FS (VAB, Caregiver reported, dressing)	24	11	2.11 (1.07)	9	2.32 (1.42)	−0.16 (−1.05, 0.72)
	FS (VAB, Caregiver reported, play)	24	11	4.51 (1.31)	9	4.69 (0.89)	−0.15 (−1.03, 0.73)
	FS (VAB, Caregiver reported, toileting)	24	11	3.03 (1.59)	9	3.48 (1.23)	−0.30 (−1.19, 0.59)

A: Low risk of bias indicates that bias is unlikely to affect the results; B: Could not be estimated due to a lack of reported estimates; C: Estimated extracted from change scores; D: Calculated SMD is not consistent with the result found in the article because of the use of change scores; HF (hand function); GM (gross motor function); and FS (functional skills).*p < 0.05.

**Figure 3:**
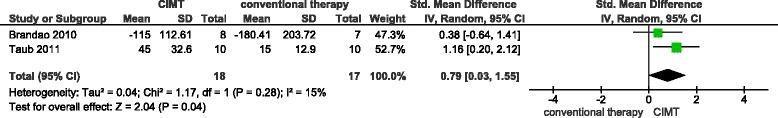
**Comparison of CIMT versus conventional therapy on unimanual hand function after 3 weeks.**

**Figure 4:**
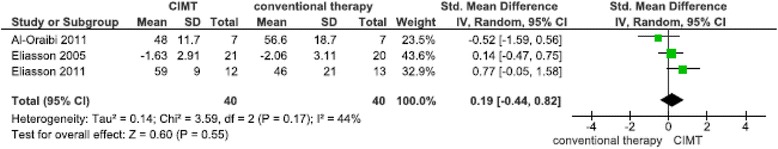
**Comparison of CIMT versus conventional therapy on bimanual hand function after 8 weeks.**

**Figure 5:**
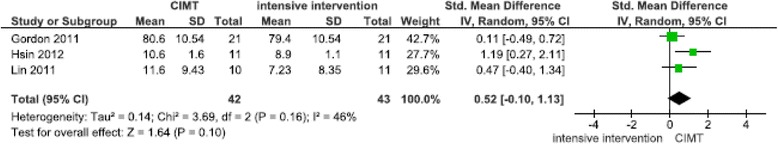
**Comparison of CIMT versus intensive interventions on unimanual hand function after 4 weeks.**

**Figure 6:**
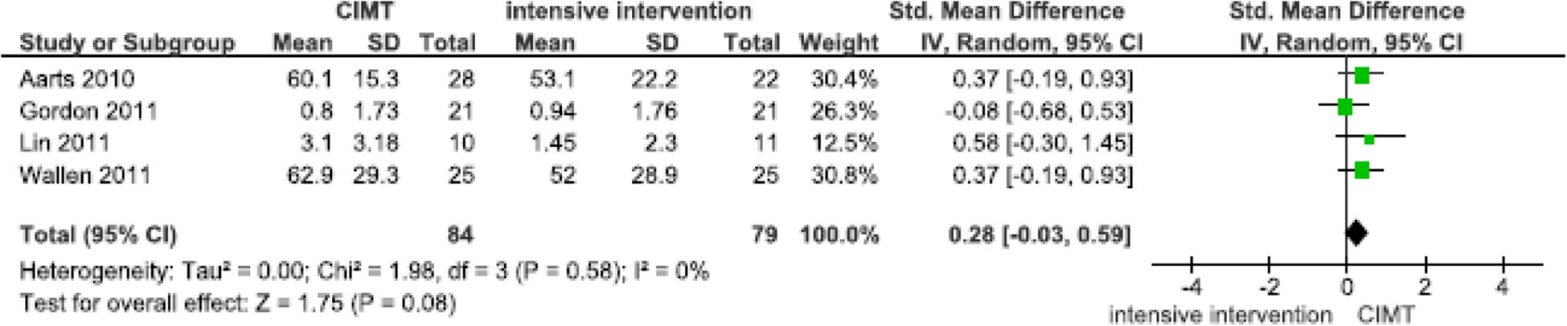
**Comparison of CIMT versus intensive interventions on bimanual hand function after 8 weeks.**

Four meta-analyses based on 10 studies that targeted hand function were performed (Figures 3, 4, 5 and 6). When compared with conventional therapy, CIMT performed for more than one hour per day showed significant effects on unilateral hand function in one meta-analysis (N = 2, [33,60] SMD 0.79 (95% CI 0.03, 1.55), p = 0.04) (Figure 3). The CIMT was practiced at the clinic [60], as home program [33,60], and incorporated into daily activities [33]. The CIMT groups performed 15–28 hours more training per week, which resulted in a difference of 29–84 training hours over two to three weeks compared with the conventional therapy groups. Unilateral hand function was assessed by the Jebsen Taylor hand function and Paediatric arm function tests. The meta-analysis was based on studies of low [60] and unclear [33] risks of bias. With regards to bimanual hand function, no significant differences were found between CIMT performed for more than one hour per day and conventional therapy (Figure 4). The CIMT was practiced as home program [19,22] or incorporated in daily activities [27]. The CIMT group had between 80–108 hours of more training compared with the conventional therapy group during the eight-week intervention period. Comparisons between CIMT performed 2–7 times per week with additional home training, or more than one hour per day at home, compared to intensive bimanual training, showed no significant findings on uni-and bimanual hand function (Figure 5, 6).

### Effects on gross motor function

The results from the 16 studies that targeted gross motor function are presented in Table 2 [39-51,55-57]. The interventions and outcomes in included studies on gross motor function were considered too heterogeneous to be pooled in meta-analyses. The results from two single studies supported the effects of intensive interventions on gross motor skills. Compared with NDT, an intensive task oriented approach performed over 3–7 sessions per week yielded higher Gross Motor Function Measure 88 (GMFM-88) scores (sitting dimension), (SMD 2.51 (CI 95% 0.63, 4.39) [40]. The task oriented intervention was performed at the clinic as individual training and without home program. Moreover, compared with CE, intensive PT and OT (control) performed over 3–7 sessions per week led to higher scores on the GMFM-88 (crawling and kneeling dimension) (SMD −1.39 (95% CI −2.56, −0.23) [55]. Both of the intensive interventions were performed at the clinic without home program. Of the 16 studies that targeted gross motor function, eight studies had less than 25 participants. All studies with significant results in favour of intensive training that targeted gross motor function had a high risk of bias (Table 2 and Figure 2). No other significant findings were observed regarding intensive interventions and gross motor function (Table 2).

## Effects on functional skills

**Figure 7:**
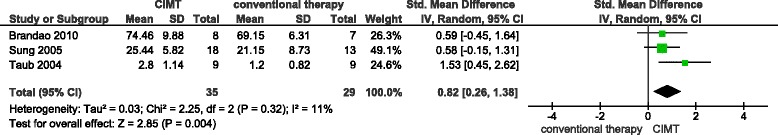
**Comparison of CIMT versus conventional therapy on functional skills after 6 weeks.**

**Figure 8:**
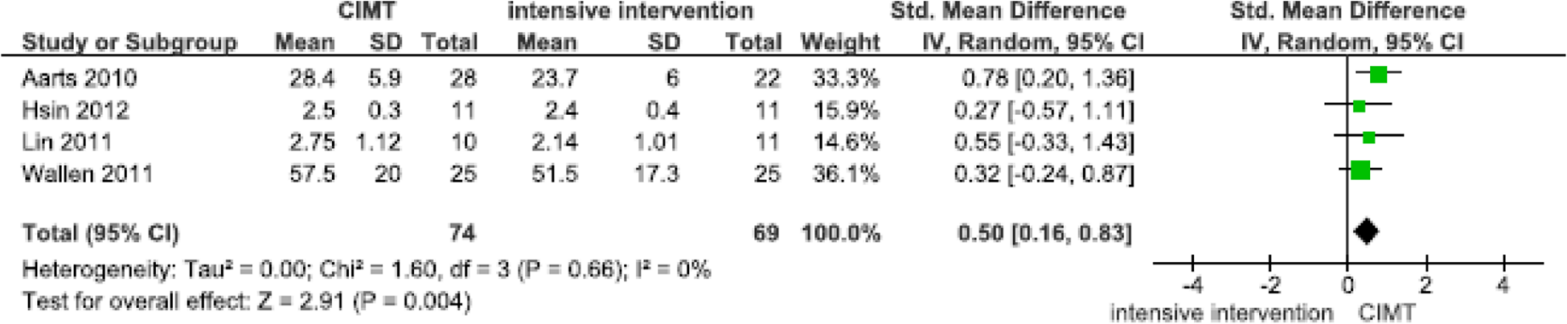
**Comparison of CIMT versus intensive interventions on functional skills after 8 weeks.**

The results from the 20 studies that targeted functional skills are presented in Table 2. Three studies measured functional skills as the only outcome [52-54], 11 studies measured functional skills in combination with hand function [21,29-38,60], three studies measured functional skills in combination with gross motor function [50,51], and three studies measured functional skills in combination with both hand function and gross motor function [55-57]. Two meta-analyses based on seven studies were performed (Figures 7, 8). CIMT was shown to affect functional skills in two meta-analyses (Figures 7, 8). The first analysis showed the following: (1) CIMT performed at least 2–7 sessions per week with additional home training achieved more improvements in functional skills compared with conventional therapy (N = 3, [36,38,60] SMD 0.82 (95% CI 0.26, 1.38), p = 0.004) (Figure 7); and (2) CIMT performed 2–7 sessions per week with additional home training achieved more improvements in functional skills compared with intensive bimanual home training (N = 4, [21,30,32,34] SMD 0.50 (95% CI 0.16, 0.83), p = 0.004) (Figure 8). Functional skills were assessed with the Paediatric Evaluation Disability Inventory (PEDI), the WeeFim, the Paediatric Motor Activity Log (PMAL), and the ABILHAND-kid. The CIMT was performed at the clinic [21,34,36,38,60], as home program [21,30,34,60] and/or in daily activities [30,32,38]. In Figure 7, the CIMT group received 15–28 hours more training per week compared with the conventional therapy group. In the second meta-analysis (Figure 8), it was not possible to calculate the differences in the amount of training between the intensive interventions and intensive control groups because the amount of home-training was not reported. One of the studies reported a difference of 24.5 hours of training per week between the intervention and control groups [32]. The first meta-analysis included two studies with a low risk of bias and one study with a high risk of bias, whereas the second meta-analysis included three studies with a low risk of bias and one study with an unclear risk of bias (Figure 2). In addition, we ran sensitivity analyses by excluding studies of high risk of bias. This did not change the significant and non-significant results.

Intensive training showed a significant effect on functional skills in two single studies [37,56]. First, compared with intensive NDT, CE performed over 3–7 sessions per week achieved greater improvements on the subscales of the Vulpe Assessment Battery (VAB), feeding, and dressing (SMD 0.85 (95% CI 0.14, 1.55); (SMD −1.26 (95% CI −2.01, −0.52)) [56] (Table 1). The CE was performed at clinic without any home program. Second, compared with regular OT, intensive NDT and casting performed over 2–7 sessions per week with additional home training led to higher improvements on the Canadian Occupational Performance Measure (COPM, satisfaction scale) (SMD 0.69 (95% CI 0.12, 1.26)) [37] (Table 2). These results were based on studies with high and unclear risks of bias [37,56] (Table 2 and Figure 2). No other significant differences were identified for functional skills (Table 2).

## Discussion

This systematic review, which included 38 studies, describes and categorises intensive motor function and functional skills training among young children with CP, and summarises the effects of these interventions. Among the studies published in the previous five years, 13 studies targeted hand function, six studies targeted gross motor functions, and 11 studies targeted functional skills. In a majority of the studies, equal improvements in motor function and functional skills were identified for intensive interventions and conventional therapy or between two different intensive interventions (Table 2).

### What works and why?

Two meta-analyses of CIMT compared with conventional therapy showed increased improvements in unilateral hand function and functional skills and were characterised by high intensity of training (Figures 3 and 7). Over a two to three week period, the CIMT groups performed 29–84 more training hours compared with the conventional therapy groups. The CIMT was performed at the clinic and home as home programs or implemented in daily activities. In contrast, when CIMT and intensive bimanual training were compared, the improvements in hand function was similar in both groups (Figures 5, 6). The included studies of these meta-analyses reported also home program and/or CIMT incorporated into daily activities. These findings suggest that in addition to different types of training the intensity of training may explain the difference in improvement between the groups, and thus be equally important as the type and context of training in determining the final outcome. These results are consistent with other systematic reviews [8,10,13]. The majority of these pooled studies had a low risk of bias, which indicates that plausible bias was unlikely to alter the results. In the non-significant meta-analysis on bimanual hand function (Figure 4), the CIMT group had 80–108 more training hours during the eight week intervention period compared with the conventional therapy group. The CIMT was practiced at the clinic and at home as home program or in daily activities. This finding is in contrasts to meta-analyses 3 and 7, and might imply that the optimal intensity is difficult to detect, which has been suggested in several systematic reviews [5-7,10,13]. As previously demonstrated, basic motor functions in young children do develop, but not at the same rate and time points as their peers [1,2]. Moreover, CIMT showed increased improvements compared with intensive bimanual training on the acquisition of functional skills (Figure 8). Three out of the four pooled studies had a low risk of bias. Eliasson [13] reported similar findings for CIMT compared with intensive bimanual training when using parent questionnaires and measures of goal-directed daily activity performance, as included in our review. However, Dong et al. [8] identified greater improvements on functional skills following intensive bimanual training compared with CIMT. A possible explanation for our finding might be the inclusion of other studies with younger participants compared with the participants included in Dong et al. [8].

Contextual factors are also important for the learning of hand function and functional skills, as they enhance the transfer of new skills to the environments where they are meant to be used [61]. We identified home training in more than 50% of the included studies. Sakzewski et al. [10] reported that 50-80% of the anticipated training dose relied on practicing at home. Home training has been found to be essential in many interventions because it can increase the total amount of training and enhance the transfer of hand function and functional skills to natural environments [10,62]. Dunst et al. [16] found that therapist-set home programs were related to decreased parent well-being and suggested that these programs might be in conflict with the families’ everyday routines and values. Additionally, he questioned whether participation in the program was actually voluntary [16]. Both Dunst [16] and Novak [62] found positive effects of home programs where parents were actively involved in goal-setting and training and received education compared with no home program. Of the 11 articles in this review that reported a significant effect of intensive interventions on motor function and functional skills [19-22,30,33,36,37,40,55,56], four studies did not have therapist-set home programs or practice incorporated in daily activities [36,40,55,56] (Tables 1 and 2). Only one study included practice incorporated in daily activities and showed inconclusive results [20,21]. In contrast, Eliasson et al. [13] noted that training and practice undertaken at home or in kindergarten tended to be below the target hours and duration. This finding might indicate that the use of a home program is less controllable and does not only increase the intensity of training but might also contribute to the variations of effects due to variable compliance [13]. To increase and maintain compliance, parents must receive adequate education and support to enable their voluntary and active involvement in goal-setting and training integrated in daily activities [16,17]. In this review, more than half of the effective interventions reported parent education and active parental involvement in training.

When comparing the training intensity (amount and duration of training periods) of studies that targeted hand function and gross motor function, the CIMT studies lasted between two and eight weeks with a high number of training sessions per week, and the gross motor interventions often lasted more than 12 weeks with fewer training sessions per week. As summarised above, some significant results were found for intensive interventions that targeted hand function. This review identified two studies [40,55] with a high risk of bias that showed effects on gross motor function, while other systematic reviews showed inconclusive results of intensive gross motor training [4,6,7]. One might ask if a higher number of training sessions per week for a shorter period practiced at home produced more motor and functional skills improvements compared with a lower number of training sessions per week for a longer period without home program. If so, this finding might suggest that the gross motor training was not sufficiently intensive and not incorporated into the child’s natural context. Some of this training might be redefined to non-intensive training, since it was performed less than one hour per session, three times per week and without home training. However, the development of gross motor function and the learning of functional skills require different physical and cognitive personal resources, as well as different contexts, and maybe different intensity than the training of hand function. This issue is unresolved and requires further investigation.

Two studies showed the effects of CE [56] and intensive NDT and casting [37] on functional skills. A high risk of bias was identified in all CE studies, whereas the intensive NDT study [37] had an unclear risk of bias, which indicated that plausible bias seriously weakens confidence in the results or raises doubt regarding the results, respectively. However, the evidence base for NDT with and without casting is conflicting [4,7]. Furthermore, Novak et al. [4] reported conflicting results for CE. Group training was present in all CE studies that targeted both motor and functional skills and seldom in CIMT that targeted hand function and functional skills. Significant effects on hand function and functional skills were identified in both interventions. One could ask whether individual training might be more effective for the learning of specific goal-directed skills, whereas group-training might be more motivational and enhance social participation. However, because the CE studies were more prone to a high risk of bias, the influence from group training on the results remains unclear. CE is offered to young children with CP, and robust research is necessary to investigate the effects.

The majority of included studies reported the activity component of ICF, and only four studies [50,55-57] reported the participation component, such as subtests of the VAB and PEDI. This is consistent with Franki et al.’s findings [5]. One possible explanation for these findings might be that activity related outcomes capture the learning of basic motor function and functional skills that are learned during young age, and that the social skills are more evident during preschool age [11].

### Limitation

Children with different subtypes of CP and functional levels might require different approaches and intensities of therapy. This aspect is not part of the scope of this review, but represents a limitation that needs to be considered when the results are interpreted. Although we have searched extensively, we may have missed relevant studies. Small studies, often without power calculations, were also included. This might indicate that some studies lacked the power to detect differences between groups. Sakzewski et al. [10] also pointed to this limitation in her review. A variety of interventions were used to improve gross motor function and functional skills, which prevented the pooling of results in the meta-analyses. Thirty-one assessment tools were used, most of which have been validated, except in studies of CE. Blinding of participants or therapists was not feasible but may still represent a risk of bias. Nineteen studies had a high risk of bias. This finding indicates that approximately half of the studies’ results are likely to be affected by bias, and therefore, the effects remain unclear.

## Conclusions

The present and other recently published systematic reviews have demonstrated the extensive and increasing evidence regarding CIMT. Studies on hand function had lower risks of bias compared with studies of gross motor function. Moreover, studies on gross motor function were typically characterised by a lower number of training sessions and longer training periods without home programs compared with studies that targeted hand function, which were characterised by higher number of training sessions and shorter training periods including home programs. These findings might suggest that more intensive training for a shorter period including practicing in the child’s natural environment may be more effective for learning functional skills. Home training appears to play an important role in increasing the intensity of training. How to implement home training without disturbing the family’s daily life in a negative manner remains to be resolved. Equal improvements in motor function and functional skills were reported for intensive interventions and conventional therapy or between two different intensive interventions in a majority of the included studies. The identification of the optimal intensity of interventions that target motor function and functional skills, as well as the possible harmful effects of intensive training, requires further investigation.

## Additional files

Additional file 1:
**Search strategy.**
Additional file 2:
**List of included studies awaiting assessment.**
Additional file 3:
**Data extraction form.**
Additional file 4:
**Characteristics of included studies.**

## References

[1] Rosenbaum PL, Walter SD, Hanna SE, Palisano RJ, Russel DJ, Raina P, Wood E, Barlett DJ, Galuppi BE (2002). Prognosis for gross motor function in cerebral palsy: creation of motor development curves. JAMA.

[2] Holmefur M, Krumlinde-Sundholm L, Bergstrøm J (2010). Longitudinal development on hand function in children with unilateral cerebral palsy. Dev Med Child Neurol.

[3] World Health Organization (2001). International Classification of Function, Disability and Health (ICF).

[4] Novak I, McIntyre S, Morgan C, Campbell L, Dark L, Morton N, Stumbles E, Wilson SA, Goldsmith S (2013). A systematic review of interventions for children with cerebral palsy: state of the evidence. Dev Med Child Neurol.

[5] Franki I, Desloovere K, De Cat J, Feys H, Molenaers G, Calders P, Vanderstraeten G, Himpens E, Van Broeck C (2012). The evidence-base for conceptual approaches and additional therapies targeting lower limb function in children with cerebral palsy: a systematic review using the ICF as a framework. J Rehabil Med.

[6] Arpino C, Vescio MF, De LA, Curatolo P (2010). Efficacy of intensive versus nonintensive physiotherapy in children with cerebral palsy: a meta-analysis. Int J Rehabil Res.

[7] Martin L, Baker R, Harvey A (2010). A systematic review of common physiotherapy interventions in school-aged children with cerebral palsy. Phys Occup Ther Pediatr.

[8] Dong VA, Tung IH, Siu HW, Fong KN (2013). Studies comparing the efficacy of constraint-induced movement therapy and bimanual training in children with unilateral cerebral palsy: a systematic review. Dev Neurorehabil.

[9] Huang HH, Fetters L, Hale J, McBride A (2009). Bound for success: a systematic review of constraint-induced movement therapy in children with cerebral palsy supports improved arm and hand use. Phys Ther.

[10] Sakzewski L, Ziviani J, Boyd RN (2014). Efficacy of upper limb therapies for unilateral cerebral palsy: a meta-analysis. Pediatrics.

[11] Haley SM, Coster WJ, Ludlow LH, Haltiwanger JT, Andrellos PJ (1992). Pediatric Evaluation of Disability Inventory: Development, Standardization, and Administration Manual, Version 1.0.

[12] Palisano RJ, Murr S (2009). Intensity of therapy services: what are the considerations?. Phys Occup Ther Pediatr.

[13] Eliasson A-C, Krumlinde-Sundholm L, Gordon A, Feys H, Klingels K, Aarts PB, Rameckers E, Autti-Rämö I, Hoare B (2014). Guidelines for future research in constraint-induced movement therapy for children with unilateral cerebral palsy: an expert consensus. Dev Med Child Neurol.

[14] Palisano RJ, Begnoche DM, Chiarello LA, Bartlett DJ, Westcott McCoy S, Chang H-J (2012). Amount and focus of physical therapy and occupational therapy for young children with cerebral palsy. Phys & Occup Ther Pediatr.

[15] Myklebust G, Jahnsen R, Elkjær S (2009). Registration of interventions in children with cerebral palsy during three years – a population based study. Dev Med Child Neurol.

[16] Dunst CJ, Bruder MB, Trivette CM, Hamby DW (2006). Everyday activity settings, natural learning environments, and early intervention practices. J Policy Pract Intellect Disabil.

[17] Novak I (2011). Parent experience of implementing effective home programs. Phys Occup Ther Pediatr.

[18] Higgins J, Green S (2008). Cochrane Handbook for Systematic Reviews of Interventions.

[19] Al-Oraibi S, Eliasson AC (2011). Implementation of constraint-induced movement therapy for young children with unilateral cerebral palsy in Jordan: a home-based model. Disabil Rehabil.

[20] Aarts PB, Jongerius PH, Geerdink YA, Van LJ, Geurts AC (2011). Modified constraint-induced movement therapy combined with bimanual training (mCIMT-BiT) in children with unilateral spastic cerebral palsy: how are improvements in arm-hand use established?. Res Dev Disabil.

[21] Aarts PB, Jongerius PH, Geerdink YA, Van LJ, Geurts AC (2010). Effectiveness of modified constraint-induced movement therapy in children with unilateral spastic cerebral palsy: a randomized controlled trial. Neurorehabil Neural Repair.

[22] Eliasson AC, Shaw K, Berg E, Krumlinde-Sundholm L (2011). An ecological approach of constraint induced movement therapy for 2-3-year-old children: a randomized control trial. Res Dev Disabil.

[23] Facchin P, Rosa-Rizzotto M, Visona Dalla PL, Turconi AC, Pagliano E, Signorini S, Tornetta L, Trabacca A, Fedrizzi E (2011). Multisite trial comparing the efficacy of constraint-induced movement therapy with that of bimanual intensive training in children with hemiplegic cerebral palsy: postintervention results. Am J Phys Med Rehabil.

[24] Gordon AM, Hung YC, Brandao M, Ferre CL, Kuo HC, Friel K, Petra E, Chinnan A, Charles JR (2011). Bimanual training and constraint-induced movement therapy in children with hemiplegic cerebral palsy: a randomized trial. Neurorehabil Neural Repair.

[25] Smania N, Aglioti SM, Cosentino A, Camin M, Gandolfi M, Tinazzi M, Fiaschi A, Faccioli S (2009). A modified constraint-induced movement therapy (CIT) program improves paretic arm use and function in children with cerebral palsy. European J Phys Rehabil Med.

[26] Charles J, Gordon AM (2006). Efficacy of hand-arm intensive bimanual training (HABIT) on upper extremity movement in children with hemiplegic cerebral palsy. Dev Med Child Neurol.

[27] Eliasson A-C, Krumlinde-Sundholm L, Shaw K, Wang C (2005). Effects of on constraint-induced movement therapy in young children with hemiplegic cerebral palsy: an adapted model. Dev Med Child Neurol.

[28] Law M, Cadman D, Rosenbaum P, Walter S, Russell D, DeMatteo C (1991). Neurodevelopmental therapy and upper-extremity inhibitive casting for children with cerebral palsy. Dev Med Child Neurol.

[29] Case-Smith J, Deluca S, Stevenson R, Ramey S (2012). A multi-center randomized controlled trial of pediatric constraint-induced movement therapy: 6-month follow-up. Am J Occup Ther.

[30] Hsin YJ, Chen FC, Lin KC, Kang LJ, Chen CL, Chen CY (2012). Efficacy of constraint-induced therapy on functional performance and health-related quality of life for children with cerebral palsy: a randomized controlled trial. J Child Neurol.

[31] Rostami HR, Malamiri RA (2012). Effect of treatment environment on modified constraint-induced movement therapy results in children with spastic hemiplegic cerebral palsy: a randomized controlled trial. Disabil Rehabil.

[32] Lin KC, Wang TN, Wu CY, Chen CL, Chang KC, Lin YC, Chen YJ (2011). Effects of home-based constraint-induced therapy versus dose-matched control intervention on functional outcomes and caregiver well-being in children with cerebral palsy. Res Dev Disabil.

[33] Taub E, Griffin A, Uswatte G, Gammons K, Nick J, Law CR (2011). Treatment of congenital hemiparesis with pediatric constraint-induced movement therapy. J Child Neurol.

[34] Wallen M, Ziviani J, Naylor O, Evans R, Novak I, Herbert RD (2011). Modified constraint-induced therapy for children with hemiplegic cerebral palsy: a randomized trial. Dev Med Child Neurol.

[35] DeLuca SC, Echols K, Law CR, Ramey SL (2006). Intensive pediatric constraint-induced therapy for children with cerebral palsy: randomized, controlled, crossover trial. J Child Neurol.

[36] Taub E, Ramey SL, Deluca S, Echols K (2004). Efficacy of constraint-induced movement therapy for children with cerebral palsy with asymmetric motor impairment. Pediatrics.

[37] Law M, Russell D, Pollock N, Rosenbaum P, Walter S, King G (1997). A comparison of intensive neurodevelopmental therapy plus casting and a regular occupational therapy program for children with cerebral palsy. Dev Med Child Neurol.

[38] Sung IY, Ryu JS, Pyun SB, Yoo SD, Song WH, Park MJ (2005). Efficacy of forced-use therapy in hemiplegic cerebral palsy. Arch Phys Med Rehabil.

[39] Carlsen PN (1975). Comparison of two occupational therapy approaches for treating the young cerebral-palsied child. Am J Occup Ther.

[40] Choi M, Lee D, Ro H (2011). Effect of task-oriented training and neurodevelopmental treatment on the sitting posture in children with cerebral palsy. J Phys Ther Sci.

[41] Kwon JY, Chang HJ, Lee JY, Ha Y, Lee PK, Kim YH (2011). Effects of hippotherapy on gait parameters in children with bilateral spastic cerebral palsy. Arch Phys Med Rehabil.

[42] Shamsoddini AR, Hollisaz MT (2009). Effect of sensory integration therapy on gross motor function in children with cerebral palsy. Iran J Child Neurol.

[43] Christiansen AS, Lange C (2008). Intermittent versus continuous physiotherapy in children with cerebral palsy. Dev Med Child Neurol.

[44] Lee JH, Sung IY, Yoo JY (2008). Therapeutic effects of strengthening exercise on gait function of cerebral palsy. Disabil Rehabil.

[45] Kanda T, Pidcock FS, Hayakawa K, Yamori Y, Shikata Y (2004). Motor outcome differences between two groups of children with spastic diplegia who received different intensities of early onset physiotherapy followed for 5 years. Brain Dev.

[46] Bower E, Michell D, Burnett M, Campbell MJ, McLellan DL (2001). Randomized controlled trial of physiotherapy in 56 children with cerebral palsy followed for 18 months. Dev Med Child Neurol.

[47] Bower E, McLellan DL, Arney J, Campbell MJ (1996). A randomised controlled trial of different intensities of physiotherapy and different goal-setting procedures in 44 children with cerebral palsy. Dev Med Child Neurol.

[48] Scherzer AL, Mike V, Ilson J (1976). Physical therapy as a determinant of change in the cerebral palsied infant. Pediatrics.

[49] Weindling AM, Cunningham CC, Glenn SM, Edwards RT, Reeves DJ (2007). Additional therapy for young children with spastic cerebral palsy: a randomised controlled trial. Health Technol Assess.

[50] Lowing K, Bexelius A, Brogren CE (2009). Activity focused and goal directed therapy for children with cerebral palsy–do goals make a difference?. Disabil Rehabil.

[51] Hur JJ-A (1997). Skills for independence for children with cerebral palsy: a comparative longitudinal study. Int J Disabil Dev Educ.

[52] de Brito BM, Gordon AM, Mancini MC (2012). Functional impact of constraint therapy and bimanual training in children with cerebral palsy: a randomized controlled trial. Am J Occup Ther.

[53] Dalvand H, Dehghan L, Feizy A, Amirsalai S, Bagheri H (2009). Effect of the Bobath technique, conductive education and education to parents in activities of daily living in children with cerebral palsy in Iran. Hong Kong J Occup Ther.

[54] McConachie H, Huq S, Munir S, Ferdous S, Zaman S, Khan NZ (2000). A randomized controlled trial of alternative modes of service provision to young children with cerebral palsy in Bangladesh. J Pediatr.

[55] Stiller C, Marcoux BC, Olson RE (2003). The effect of conductive education, intensive therapy, and special education services on motor skills in children with cerebral palsy. Phys Occup Ther Pediatr.

[56] Reddihough DS, King J, Coleman G, Catanese T (1998). Efficacy of programmes based on Conductive Education for young children with cerebral palsy. Dev Med Child Neurol.

[57] Coleman GJ, King JA, Reddihough DS (1995). A pilot evaluation of conductive education-based intervention for children with cerebral palsy: the Tongala project. J Paediatr Child Health.

[58] Eliasson AC, Krumlinde-Sundholm L, Rosblad B, Beckung E, Arner M, Ohrvall AM, Rosenbaum P (2006). The Manual Ability Classification System (MACS) for children with cerebral palsy: scale development and evidence of validity and reliability. Dev Med Child Neurol.

[59] Palisano R, Rosenbaum P, Walter S, Russell D, Wood E, Galuppi B (1997). Development and reliability of a system to classify gross motor function in children with cerebral palsy. Dev Med Child Neurol.

[60] Brandao M, Mancini MC, Vaz DV, de Melo APP, Fonseca ST (2010). Adapted version of constraint-induced movement therapy promotes functioning in children with cerebral palsy: a randomized controlled trial. Clin Rehabil.

[61] Shumway-Cook A, Wollacot MH (2007). Motor Control: Translating Research into Clinical Practice.

[62] Novak I, Cusick A, Lannin N (2009). Occupational therapy home programs for cerebral palsy: double-blind, randomized, controlled trial. Pediatrics.

