# Supplementary material for: Intensive training of motor function and functional skills among young children with cerebral palsy: a systematic review and meta-analysis
Source: BMC Pediatr. 2014 Dec 5;14:292. doi: 10.1186/s12887-014-0292-5 (PMC4265534; doi:10.1186/s12887-014-0292-5)
Supplement: Additional file 1: — Search strategy. [file 12887_2014_292_MOESM1_ESM.pdf]

Appendix I. Search strategy Ovid MEDLINE(R)

Database: Ovid MEDLINE(R) In-Process & Other Non-Indexed Citations and Ovid MEDLINE(R) <1946 to Present>

10.10.2012

----

1 exp Child/ (3158363)

2 exp Infant/ (1402417)

3 exp Newborn/ (949581)

4 (child$ or pediatric$ or paediatric$ or boy$1 or girl$1 or kid$1 or preschool$ or juvenil$ or under?age$ or minor$ or infant$ or baby or babies or toddler$ or newborn$).tw. (4152623)

5 or/1-4 (5899428)

6 Cerebral Palsy/ (40462)

7 ((cerebr$ adj2 pals$) or neuro-disab$ or neurodisab$).tw. (37173)

8 ("little's disease" or "little disease" or (spastic adj2 diplegia$)).tw. (1977)

9 or/6-8 (48801)

10 exp Exercise Therapy/ (69083)

11 Movement Therapy/ (2435)

12 exp kinesiotherapy/ (42672)

13 exp Exercise/ (298533)

14 exercises.tw. (56340)

15 ((Physical or therap*) adj1 exercis*).tw. (30789)

16 (activit* adj focus*).tw. (1026)

17 virtual reality/ (10267)

18 Computer Simulation/ (200451)

19 (virtual realit* or (computer adj2 simulat*)).tw. (51280)

20 (adapt$ adj (physical activit$ or sport$)).tw. (312)

21 massag$.tw. (16782)

22 ((intensive or functional$ or goal-directed or goal directed or task-oriented or task oriented or neurodevelopment$ or occupational or intensive) adj2 (therapy or therapies or train$ or treatment$ or stimulat$ or program$ or intervention$)).tw. (97810)

23 kinesiotherap$.tw. (358)

24 (peto or "move and walk").tw. (1740)

25 (conductive adj (educat$ or pedagog$)).tw. (194)

26 (aquatic$ or swim$).tw. (106688)

27 treadmill$.tw. (48617)

28 (family hope or craniosacral or cranio sacral or fascia release or brain therapy).tw. (405)

29 (kozijavskin$ or koziavkin$ or kozijavkin$ or koziavskin$ or intensive neurophysiological rehabilitation).tw. (6)

30 atoh.tw. (25)

31 ((family centered adj service$) or (family centred adj service$)).tw. (319)

32 doman$.mp. or (iahp or achievement of human potential).tw. (834)

33 vojta$.tw. (268)

34 (deinstitutionali$ or (activit$ adj2 daily living) or adl or independent living or self care).tw. (78052)

35 Activities of Daily Living/ (100078)

36 "Activities of Daily Living"/ (100078)

37 Daily Life Activity/ (49791)

38 exp daily activities/ (2379)

39 exp self care skills/ (3063)

40 Occupational Therapy/ (32802)

41 exp Physical Therapy Modalities/ (162424)

42 exp physiotherapy/ (49374)
43 physiotherap$.tw. (36743)
44 physical therap$.tw. (30174)
45 ergotherap$.tw. (831)
46 ergo therap$.tw. (23)
47 or/10-46 (1144924)
48 (comment or letter or editorial).pt. (2429971)
49 5 and 9 and 47 (6073)
50 49 not 48 (5922)
51 (study or studies or trial* or impact* or evaluat* or assess* or group* or research* or outcome* or effect* or efficacy or compar* or experiment* or control* or random* or cohort* or case-control* or observational* or empirical*).tw. (27887913)
52 (clinical trial or comparative study or evaluation studies or meta analysis or multicenter study or randomized controlled trial).pt. (2261731)
53 (clinical case study or empirical study or experimental replication or field study or followup study or longitudinal study or meta analysis or nonclinical case study or prospective study or quantitative study or retrospective study or "systematic review" or treatment outcome clinical trial or twin study).md. (1582759)
54 51 or 52 or 53 (28421613)
55 50 and 54 (4792)
56 limit 50 to "reviews (best balance of sensitivity and specificity)" (879)
57 55 or 56 (4905)
58 remove duplicates from 57 (3270)
