# Supplementary material for: Intensive training of motor function and functional skills among young children with cerebral palsy: a systematic review and meta-analysis
Source: BMC Pediatr. 2014 Dec 5;14:292. doi: 10.1186/s12887-014-0292-5 (PMC4265534; doi:10.1186/s12887-014-0292-5)
Supplement: Additional file 2: — List of included studies awaiting assessment. [file 12887_2014_292_MOESM2_ESM.docx]

**Appendix II. List of included studies awaiting assessment**

1. [Abd El-Kafy EM](http://www.ncbi.nlm.nih.gov.proxy.helsebiblioteket.no/pubmed?term=Abd%20El-Kafy%20EM%5BAuthor%5D&cauthor=true&cauthor_uid=24325594), [Elshemy SA](http://www.ncbi.nlm.nih.gov.proxy.helsebiblioteket.no/pubmed?term=Elshemy%20SA%5BAuthor%5D&cauthor=true&cauthor_uid=24325594), [Alghamdi MS](http://www.ncbi.nlm.nih.gov.proxy.helsebiblioteket.no/pubmed?term=Alghamdi%20MS%5BAuthor%5D&cauthor=true&cauthor_uid=24325594): **Effect of constraint-induced therapy on upper limb functions: a randomized control trial.** [*Scand J Occup Ther.*](http://www.ncbi.nlm.nih.gov.proxy.helsebiblioteket.no/pubmed?term=((Abd%20El-Kafy%20EM)%20AND%20upper%20limb%20functions)%20AND%202014) 2014, **21**:11-23. doi: 10.3109/11038128.2013.837505. Epub 2013 Dec 11.
2. [Choudhary A](http://www.ncbi.nlm.nih.gov.proxy.helsebiblioteket.no/pubmed?term=Choudhary%20A%5BAuthor%5D&cauthor=true&cauthor_uid=23238223), [Gulati S](http://www.ncbi.nlm.nih.gov.proxy.helsebiblioteket.no/pubmed?term=Gulati%20S%5BAuthor%5D&cauthor=true&cauthor_uid=23238223), [Kabra M](http://www.ncbi.nlm.nih.gov.proxy.helsebiblioteket.no/pubmed?term=Kabra%20M%5BAuthor%5D&cauthor=true&cauthor_uid=23238223), [Singh UP](http://www.ncbi.nlm.nih.gov.proxy.helsebiblioteket.no/pubmed?term=Singh%20UP%5BAuthor%5D&cauthor=true&cauthor_uid=23238223), [Sankhyan N](http://www.ncbi.nlm.nih.gov.proxy.helsebiblioteket.no/pubmed?term=Sankhyan%20N%5BAuthor%5D&cauthor=true&cauthor_uid=23238223), [Pandey RM](http://www.ncbi.nlm.nih.gov.proxy.helsebiblioteket.no/pubmed?term=Pandey%20RM%5BAuthor%5D&cauthor=true&cauthor_uid=23238223), [Kalra V](http://www.ncbi.nlm.nih.gov.proxy.helsebiblioteket.no/pubmed?term=Kalra%20V%5BAuthor%5D&cauthor=true&cauthor_uid=23238223): **Efficacy of modified constraint induced movement therapy in improving upper limb function in children with hemiplegic cerebral palsy: a randomized controlled trial.** [*Brain Dev*](http://www.ncbi.nlm.nih.gov.proxy.helsebiblioteket.no/pubmed?term=((Choudhary%20A)%20AND%20children%20with%20hemiplegic%20cerebral%20palsy)%20AND%202013) 2013, **35**:870-6. doi: 10.1016/j.braindev.2012.11.001. Epub 2012 Dec 11.
3. [Deppe W](http://www.ncbi.nlm.nih.gov.proxy.helsebiblioteket.no/pubmed?term=Deppe%20W%5BAuthor%5D&cauthor=true&cauthor_uid=23818409), [Thuemmler K](http://www.ncbi.nlm.nih.gov.proxy.helsebiblioteket.no/pubmed?term=Thuemmler%20K%5BAuthor%5D&cauthor=true&cauthor_uid=23818409), [Fleischer J](http://www.ncbi.nlm.nih.gov.proxy.helsebiblioteket.no/pubmed?term=Fleischer%20J%5BAuthor%5D&cauthor=true&cauthor_uid=23818409), [Berger C](http://www.ncbi.nlm.nih.gov.proxy.helsebiblioteket.no/pubmed?term=Berger%20C%5BAuthor%5D&cauthor=true&cauthor_uid=23818409), [Meyer S](http://www.ncbi.nlm.nih.gov.proxy.helsebiblioteket.no/pubmed?term=Meyer%20S%5BAuthor%5D&cauthor=true&cauthor_uid=23818409), [Wiedemann B](http://www.ncbi.nlm.nih.gov.proxy.helsebiblioteket.no/pubmed?term=Wiedemann%20B%5BAuthor%5D&cauthor=true&cauthor_uid=23818409): **Modified constraint-induced movement therapy versus intensive bimanual training for children with hemiplegia - a randomized controlled trial.** [*Clin Rehabil.*](http://www.ncbi.nlm.nih.gov.proxy.helsebiblioteket.no/pubmed?term=((Deppe%20W)%20AND%20children%20with%20hemiplegia)%20AND%202013) 2013, **27**:909-20. doi: 10.1177/0269215513483764. Epub 2013 Jul 1.
4. [Gelkop N](http://www.ncbi.nlm.nih.gov.proxy.helsebiblioteket.no/pubmed?term=Gelkop%20N%5BAuthor%5D&cauthor=true&cauthor_uid=24983295), [Burshtein DG](http://www.ncbi.nlm.nih.gov.proxy.helsebiblioteket.no/pubmed?term=Burshtein%20DG%5BAuthor%5D&cauthor=true&cauthor_uid=24983295), [Lahav A](http://www.ncbi.nlm.nih.gov.proxy.helsebiblioteket.no/pubmed?term=Lahav%20A%5BAuthor%5D&cauthor=true&cauthor_uid=24983295), [Brezner A](http://www.ncbi.nlm.nih.gov.proxy.helsebiblioteket.no/pubmed?term=Brezner%20A%5BAuthor%5D&cauthor=true&cauthor_uid=24983295), [Al-Oraibi S](http://www.ncbi.nlm.nih.gov.proxy.helsebiblioteket.no/pubmed?term=Al-Oraibi%20S%5BAuthor%5D&cauthor=true&cauthor_uid=24983295), [Ferre CL](http://www.ncbi.nlm.nih.gov.proxy.helsebiblioteket.no/pubmed?term=Ferre%20CL%5BAuthor%5D&cauthor=true&cauthor_uid=24983295), [Gordon AM](http://www.ncbi.nlm.nih.gov.proxy.helsebiblioteket.no/pubmed?term=Gordon%20AM%5BAuthor%5D&cauthor=true&cauthor_uid=24983295): **Efficacy of Constraint-Induced Movement Therapy and Bimanual Training in Children with Hemiplegic Cerebral Palsy in an Educational Setting.** [*Phys Occup Ther Pediatr.*](http://www.ncbi.nlm.nih.gov.proxy.helsebiblioteket.no/pubmed?term=((Gelkop%20N)%20AND%20children%20with%20hemiplegic%20cerebral%20palsy)%20AND%202014) 2014. [Epub ahead of print]
5. [Mahasup N](http://www.ncbi.nlm.nih.gov.proxy.helsebiblioteket.no/pubmed?term=Mahasup%20N%5BAuthor%5D&cauthor=true&cauthor_uid=23964461), [Sritipsukho P](http://www.ncbi.nlm.nih.gov.proxy.helsebiblioteket.no/pubmed?term=Sritipsukho%20P%5BAuthor%5D&cauthor=true&cauthor_uid=23964461), [Lekskulchai R](http://www.ncbi.nlm.nih.gov.proxy.helsebiblioteket.no/pubmed?term=Lekskulchai%20R%5BAuthor%5D&cauthor=true&cauthor_uid=23964461), [Hansakunachai T](http://www.ncbi.nlm.nih.gov.proxy.helsebiblioteket.no/pubmed?term=Hansakunachai%20T%5BAuthor%5D&cauthor=true&cauthor_uid=23964461): **Effects of mirror neurons stimulation on motor skill rehabilitation in children with cerebral palsy: a clinical trial.** [*J Med Assoc Thai.*](http://www.ncbi.nlm.nih.gov.proxy.helsebiblioteket.no/pubmed?term=((Mahasup%20N)%20AND%20children%20with%20cerebral%20palsy)%20AND%202012) 2012, **95**:S166-72.
6. Mattern-Baxter K, McNeil S, Mansoor JK: [**Effects of home-based locomotor treadmill training on gross motor function in young children with cerebral palsy: a quasi-randomized controlled trial.**](http://www.ncbi.nlm.nih.gov.proxy.helsebiblioteket.no/pubmed/23747646) *Arch Phys Med Rehabil.* 2013, **94**:2061-7. doi: 10.1016/j.apmr.2013.05.012. Epub 2013 Jun 5.
7. [Shamir M](http://www.ncbi.nlm.nih.gov.proxy.helsebiblioteket.no/pubmed?term=Shamir%20M%5BAuthor%5D&cauthor=true&cauthor_uid=23393711), [Dickstein R](http://www.ncbi.nlm.nih.gov.proxy.helsebiblioteket.no/pubmed?term=Dickstein%20R%5BAuthor%5D&cauthor=true&cauthor_uid=23393711), [Tirosh E](http://www.ncbi.nlm.nih.gov.proxy.helsebiblioteket.no/pubmed?term=Tirosh%20E%5BAuthor%5D&cauthor=true&cauthor_uid=23393711): **Intensive intermittent physical therapy in infants with cerebral palsy: a randomized controlled pilot study.** [*Isr Med Assoc J.*](http://www.ncbi.nlm.nih.gov.proxy.helsebiblioteket.no/pubmed?term=((Shamir%20M)%20AND%20infants%20with%20cerebral%20palsy)%20AND%202012) 2012, **14**:737-41.
