# Supplementary material for: Intensive training of motor function and functional skills among young children with cerebral palsy: a systematic review and meta-analysis
Source: BMC Pediatr. 2014 Dec 5;14:292. doi: 10.1186/s12887-014-0292-5 (PMC4265534; doi:10.1186/s12887-014-0292-5)
Supplement: Additional file 3: — Data extraction form. [file 12887_2014_292_MOESM3_ESM.pdf]

Appendix III. "Data extraction «Intensive training- a systematic review"

Primary author, year: **Country:**

Reviewer: **Date:**

Aim of the study:

Study population

	All participants	Intervention group	Comparator (1)	Comparator (2)
N (number of participants)				
Age (specify)				
Gender (Femal/Male)				
CP spastic unilateral (left/right)				
CP spastic bilateral				
CP dyskinetic				
CP ataxic				
GMFCS level I-V				
MACS level I-V				
Other functional descriptions				
Additional CP related impairments				

Comments:
Inclusion criteria:
Exclusion criteria:

Study interventions
Type of intervention(s) described by authors (short description):

Intervention (I):

Comparator (C1):

Comparator (C2):

□ Intervention directed towards child, be specific:

□ Interventions directed towards environment, be specific:

Setting

	Intervention	Comparator (1)	Comparator (2)
Home			
Kindergarten/school			
Clinic/center			
Other			

Comments:

Organisation of interventions

	Intervention	Comparator (1)	Comparator (2)
Individual			
Group			
Incorporated into daily activities			

Comments:

Involvement in training (describe the role; observer, facilitator, or performer)

	Intervention (observer/facilitator, performed)	Comparator (1) (observer/facilitator, performed)	Comparator (2) (observer/facilitator, performed)
Parents			
Professionals			
Others			
Supervision of (y/n)			
Supervision of others			

Comments:

Amount of intervention (describe the amount of training performed not according to protocol)

	Intervention	Comparator (1)	Comparator (2)
Duration of inter-vention (wk/mo)			
Frequency of session/ wk or mo			
Duration of session (min/h)			
Hours/ week			
Frequency of task practicing (home/kinder)			
Hours/week			

Comments:

Use of goals

□ Not reported	□ Yes	Intervention	Comparator (1)	Comparator (2)
	General goals			
	Specific goals			
	Parent set			
	Therapist set			
	Shared goals			

Comments:

Outcome (activity and participation)

**Outcome**	**Measurement scale**	**Scale range**
**1.**		
**2.**		
**3.**		
**4.**		

Comments (mention other outcomes described in the study):
□ **Drop out, describe:**

Results (present the results in the same order as the outcomes)

	Intervention	Int n	Comparator (1)	Com1 n	Comparator (2)	Comp2 n
Outcome						
Measurement scale						
Baseline score						
Post treatment 1 (SD, p-value)						
Post- treatment 2 (SD, p-value)						
Post- treatment 3 (SD, p-value)						
Effect size- mean difference, adjusted diff (SD/SE)						
Effect size (CI, p-value)						

Comments (as adjusted for):
□ No adverse effect reported
□ Adverse effects, type
□ Compliance
