# Supplementary material for: Intensive training of motor function and functional skills among young children with cerebral palsy: a systematic review and meta-analysis
Source: BMC Pediatr. 2014 Dec 5;14:292. doi: 10.1186/s12887-014-0292-5 (PMC4265534; doi:10.1186/s12887-014-0292-5)
Supplement: Additional file 4: — Characteristics of included studies. [file 12887_2014_292_MOESM4_ESM.docx]

**Appendix IV. Characteristics of included studies**

| **Author, year,**  **country**  **study design, Risk of bias (ROB)** | **Outcome; HF** (hand function), **GM**  (gross motor function), **FS** (functional skills)  **(outcome measurements)** | **Intervention** | **Comparator (s)** | **Participants**  **N, Subtypes of CP***  **Mean age (years, months), SD** |
| --- | --- | --- | --- | --- |
| Al-Oraibi, 2011,  Jordan  RCT, high ROB | **HF** (AHA) | CIMT and home program | NDT | N=20, spastic unilateral CP  Mean age (I): 3y 9mo (1y 6mo)  Mean age (C): 5y 4mo (2y 2mo) |
| Aarts 2011,  and 2010  Netherlands  RCT, low ROB | **HF** (VOAA-DDD) | CIMT and BiT at home | PT/OT sessions and intensive bimanual training at home | N=52, spastic unilateral CP  Mean age (I): 4y 8mo (1y 3mo)  Mean age (C):5y 1mo (1y 7mo) |
|  | **HF, FS** (AHA, ABILHAND-kid) | CIMT and BiT at home | PT/OT sessions and intensive bimanual training at home |  |
| Eliasson, 2011,  Sweden  Cross-over, low ROB | **HF**(AHA) | Eco-CI and conventional pediatric treatment | Conventional pediatric treatment (PT/OT) | N=25, spastic unilateral CP  Mean age: 2y 4mo (0y 9mo) |
| Facchin 2011,  Italy  RCT, unclear ROB | **HF** (QUEST, Besta Scale, quality of grasp) | mCIMT and home practicing | - Bimanual intensive rehabilitation treatment (IRP)  - Standard care (ST) | N=105, spastic unilateral CP  Mean age (CIMT): 3y 0mo (0y 4mo)  Mean age (IRP): 2y 9mo (0y 7mo)  Mean age (ST): 3y 1mo (0y 4mo) |
| Gordon, 2011,  USA  RCT, low ROB | **HF**(JTTHF, AHA, QUEST) | CIMT (90 hours) and home program | HABIT (90 hours) and home program | N=44, spastic unilateral CP  Mean age (CIMT): 6y 3mo (2y 2mo)  Mean age (HABIT): 6y 4mo (1y 1mo) |
| Smania, 2009,  Italia  cross over, high ROB | **HF** (Use Test, Function Test) | mCIT and home practicing | Conventional PT | N=10, spastic unilateral CP  Mean age: 3y 3mo (NR) |
| Charles, 2006 ,  USA  cross-over trial, unclear ROB | **HF** (JTTHF, BOTMP, subtest 8) | CIMT and home program and treatment as usual | Waiting list with treatment as usual | N=66, spastic unilateral CP  Mean age: 6y 8mo (1y 4mo) |
| Eliasson, 2005,  Sweden  CBA, high ROB | **HF** (AHA) | CI and conventional pediatric treatment | Conventional pediatric treatment | N=41, spastic unilateral CP  Mean age (I): 2y 4mo (0y 7 mo)  Mean age (C): 2y 6mo (0y 9mo) |
| Law, 1991,  Canada  RCT, unclear ROB | **HF** (PDMS, Fine Motor Scale  QUEST) | Intensive NDT and casting | Regular NDT and casting  -Intensive NDT  -Regular NDT | N=73, spastic uni- and bilateral CP  Mean age: NR  Age: 18 months -8 yr |
| Case- Smith, 2012,  USA  RCT, low ROB | **HF, FS** (AHA, QUEST, PMAL) | CIMT (3 hours/day) | CIMT (6 hours/day) | N=18, spastic unilateral CP  Mean age: 4y 1mo (1y 0mo) |
| Hsin, 2012,  Taiwan  RCT, low ROB | **HF, FS** (BOTMP subtest 8, PMAL) | CI (home) and home practicing | NDT and functional oriented activities | N=23, spastic unilateral CP  Mean age (I): 6y 9mo (0y 6mo)  Mean age (c): 6y 9mo (0y 6mo) |
| Rostami, 2012,  Iran  RCT, unclear ROB | **HF, FS** (BOTMP subtest 8, PMAL) | mCIMT (home) | mCIMT (clinic) | N=14, spastic unilateral CP  Mean age: 6y 2mo (1y 3mo) |

| Lin, 2011,  Taiwan  RCT, high ROB | **HF, FS** (PDMS-2, BOTMP, subtest 8, PMAL) | Home-based CI therapy | Home-based functional unilateral or bilateral arm training, including restraint | N=21, spastic uni-and bilateral CP  Mean age (I): 6y 4mo (2y 2mo)  Mean age (C): 6y 9mo (2y1mo) |
| --- | --- | --- | --- | --- |
| Taub, 2011,  USA  cross over trial, unclear ROB | **HF, FS** (INMAP, PAFT, PMAL ) | CI | Conventional therapy (PT/OT) | N=20, spastic unilateral CP  Mean age (I): 4y 0mo (1y 2mo)  Mean age (C): 3y 3mo (1y 6mo) |
| Wallen, 2011,  Australia  RCT, low ROB | **HF, FS** (AHA, COPM, PMAL) | CI | Intensive goal-directed OT | N=50, spastic unilateral CP  Mean age: 4y 1mo (1y 8mo) |
| DeLuca, 2006,  USA  Cross over trail, unclear risk  Taub, 2004 ,  USA  RCT, low ROB | **HF, FS** (QUEST, EBS, PMAL) | CIT | Usual care (PT and OT) | N=18, spastic unilateral CP  Mean age: 3y 5mo (NR) |
|  | **HF, FS** (EBS, TAUT, PMAL) | CI | Conventional therapy (PT and OT) |  |
| Law, 1997,  Canada  cross over trial, unclear ROB | **HF, FS** (PDMS, Fine Motor Scale  QUEST, COPM) | Intensive NDT and casting | Regular OT | N=52, spastic uni-and bilateral CP  Mean age: 2y 7mo (0y 9mo) |
| Brandao, 2010 ,  USA  RCT, low ROB | **HF, FS** (JTTHF, PEDI) | CIMT followed by bimanual functional training at home | OT (Bimanual activities and sensory stimulation) | N=16, spastic unilateral CP  Mean age (I): 5y 6mo (NR)  Mean age (C): 6y 7mo (NR) |
| Sung, 2005,  South-Korea  RCT, high ROB | **HF, FS** (BBT, EDPA, WeeFIM) | CI and conventional OT and home practicing (FUT) | Conventional OT | N=31, spastic unilateral CP  Mean age (I): 2y 8mo (0y 7mo)  Mean age (C): 3y 6 mo (2y 3mo) |
| Carlsen, 1975,  USA  CBA, high ROB | **HF, GM** (Denver Development subscales) | OT facilitation treatment (NDT) | OT functional treatment | N=20, spastic uni- and bilateral Mean age: 2y 10mo (NR) |
| Choi, 2011,  Korea  RCT, high ROB | **GM** (GMFM-88, Dimension B, Sitting) | Task oriented approach | NDT | N=10, spastic bilateral CP  Mean age (I): 3y 4mo (NR)  Mean age (C): 4y 0mo (NR) |
| Kwon, 2010,  Korea  CBA, high ROB | **GM** (GMFM-88, dimensions D (standing) and E (walking, running, jumping)) | Hippotheraphy and NDT | NDT | N=32, spastic bilateral CP  Mean age (I): 6y 1mo (1y 7mo)  Mean age (C): 6y 4mo (1y 7mo) |
| Shamsoddini, 2009,  Iran  RCT, high ROB | **GM** (GMFM-88) | Sensory Integration therapy | Intensive home program (routine OT performed by parents and supervised by an OT) | N=24, spastic bilateral CP  Mean age 3y 4mo (NR) |
| Christiansen 2008,  Denmark  RCT, unclear ROB | **GM** (GMFM-66) | Intermitted PT | Continuous PT | N=25, CP  Age: 1-8 y  Mean age: NR |
| Lee, 2008,  Korea  RCT, high ROB | **GM** (GMFM-88, Computerized gait analysis) | Strengthening program | Conventional PT | N=17, spastic uni-and bilateral CP  Mean age (I): 6y 3mo (2y 1mo)  Mean age (C): 6y 3mo (2y 9mo) |
| Kanda, 2004,  Japan  CBA, high ROB | **GM** (Abel to stand 5 sec or walk) | Sufficient Vojta program (FT) | No training or insufficient Vojta program (IT) | N=10, spastic bilateral CP Age: Mean age: 0y 6mo (NR) |
| Bower 2001,  UK  RCT, low ROB | **GM** (GMFM-88) | Intensive PT and goals | - PT and goals  -Intensive PT and aims  - PT and aims | N=56, spastic bilateral CP  Mean age (I): 5y 5 mo (NR)  Mean age (C1): 5y 9 mo (NR)  Mean age (C2): 5y 4 mo (NR)  Mean age (C3): 6y 3mo (NR) |
| Bower, 1996,  UK  RCT, unclear ROB | **GM** (GMFM-88) | Intensive PT and goals | PT and goals  -Intensive PT and aims  -PT and aims | N=44, spastic bilateral CP  Mean age: NR  Age: 3-11y |
| Scherzer, 1976,  USA  RCT, high ROB | **GM** (Motor Development Evaluation Form) | Home program to facilitate motor development. | Traditional passive range of motion exercises | N=24, spastic uni-and bilateral, dyskinetic, and ataxic CP  Mean age: 0y 10mo (NR) |
| Weindling, 2007,  UK  RCT, low ROB | **GM, FS** (GMFM-88, Vineland, daily living) | Standard PT and extra PT from a PT assistant (PAG) | - Standard PT (CG)  - Standard PT and visit by a family support worker (FSWG) | N=88, spastic uni-and bilateral CP  Mean age: 1y 7mo (0y 7mo) |
| Løwing, 2009,  Sweden  CBA, high ROB | **GM, FS** (GMFM-66, PEDI) | Goal-directed functional training (GT) | Activity-focused therapy | N=44, spastic uni-and bilateral CP  Mean age: 4y 1mo (1y 5mo) |
| Hur, 1997,  UK  CBA, high ROB | **GM, FS** (VAB, Developmental Profile 2) | CE | British special education program | N=40, CP  Mean age: NR  Age: 3.5-4.5 yr |
| Brandao 2012,  USA  RCT, unclear ROB | **FS** (PEDI, COPM) | 90 hours CIMT and home program | 90 hours HABIT and home program | N=16, spastic unilateral CP  Mean age: 6y 3mo (NR) |
| Dalvand, 2009,  Iran  CBA, high ROB | **FS** (CDER) | CE | -NDT  -Education to parents | N=45, spastic uni- and bilateral and dystonic CP  Mean age (I): 5y 9mo (1y 4mo)  Mean age (C1):5y 7mo (1y 4mo)  Mean age (C2): 6y 1mo (1y 4mo) |
| McConahie, 2000,  Bangladesh  RCT, high ROB | **FS** (IBAS) | Urban daily mother-  child group | - Urban monthly distance training  - Rural monthly distance training  - Rural health advice | N=85, spastic uni- and bilateral and dyskinetic CP  Mean age (I): 2y 7mo (1y 0mo)  Mean age (C1): 3y 0mo(0y8mo)  Mean age (C2): 3y 7mo(1y1mo)  Mean age (C3): 3y 3mo (1y3mo) |
| Stiller, 2003,  USA  CBA, high ROB | **HF, GM, FS** (PDMS, Fine Motor Scale, GMFM-88, PEDI) | CE | -Intensive individual and group OT and PT (IT)  - Intensive special education | N=21, spastic uni-and bilateral CP  Mean age (I):6y 3mo (1y8mo)  Mean age (C1):3y 10mo (1y7mo)  Mean age (C2): 3y 9mo (0y 9mo) |
| Reddihough, 1998,  Australia  RCT, high ROB | **HF, GM, FS** (GMFM-88, VAB) | CE individual and in group | Intensive neuro-developmental programs | N=34, spastic bilateral and ataxic CP  Mean age: 1y 9mo (NR) |
| Coleman, 1995,  Australia  CBA, high ROB | **HF, GM, FS** (VAB) | CE | Traditional early intervention program | N=20, spastic uni- and bilateral Mean age: 3y 8mo (NR) |

*Classification of subtype of CP according to Surveillance of Cerebral palsy in Europe (SCPE) (Cans et al 2007)

AHA (Assisting Hand Assessment), APCP (Assessment of Preschool Children’s Participation), BBT (Box and block test), BiT (Bimanual Intensive Training), BOTMP subtest 8 (Bruininks–Oseretsky Test of Motor subtest 8),

CI (Constraint Induced), CE (Conductive Education), CDER (The client development evaluation report), CIMT (Constraint Induced Movement Therapy), COPM (Canadian Occupational Performance Measure),

EBS (Emerging behaviors scale), EDPA (Erhardt Developmental Prehension Assessment), GMFM (Gross Motor Function Measures), HABIT (hand-arm intensive bimanual therapy), IBAS (Independent behaviour Assessment Scale),

INMAP ( Pediatric arm function test Inventory of new motor activities and programs instrument), JTTHF (Jebsen-Taylor test of hand function), mCIMT (modified Constraint Induced Movement Therapy), NDT (Neurodevelopmental therapy), NR (not reported) OT (Occupational Therapy), PDMS (Peabody Development Motor Scales, Fine Motor Scale ), PEDI (Pediatric Evaluation of Disability Inventory, PMAL (Pediatric Motor Activity Log), PMAL AOU (Amount of Hand Use subscale), PMAL QOU (Quality of Hand Use subscale), PT (Physiotherapy), QUEST (Quality of Upper Extremity Skills Test), TAUT (Toddler Arm Use test), VOAA-DDD (Video observation Arts and Arts - capacity, performance, and developmental disregard), VAB (Vulpe Assessment Battery).
